# Association between self-reported caffeine intake during pregnancy and social responsiveness scores in childhood: The EARLI and HOME studies

**DOI:** 10.1371/journal.pone.0245079

**Published:** 2021-01-15

**Authors:** Marisa A. Patti, Nan Li, Melissa Eliot, Craig Newschaffer, Kimberly Yolton, Jane Khoury, Aimin Chen, Bruce P. Lanphear, Kristen Lyall, Irva Hertz-Picciotto, Margaret Daniele Fallin, Lisa A. Croen, Joseph M. Braun

**Affiliations:** 1 Department of Epidemiology, Brown University, Providence, Rhode Island, United States of America; 2 A.J. Drexel Autism Institute, Drexel University, Philadelphia, Pennsylvania, United States of America; 3 College of Health & Human Development, Pennsylvania State University, State College, Pennsylvania, United States of America; 4 Department of Pediatrics, Cincinnati Children’s Hospital Medical Center, Cincinnati, Ohio United States of America; 5 Department of Pediatrics, University of Cincinnati College of Medicine, Cincinnati, Ohio, United States of America; 6 Department of Health Sciences, Simon Fraser University, British Columbia, Vancouver, Canada; 7 Department of Public Health Sciences, University of California, Davis, California, United States of America; 8 Department of Mental Health, Johns Hopkins University, Baltimore, Maryland, United States of America; 9 Division of Research, Kaiser Permanente Northern California, Oakland, California, United States of America; Stony Brook University, Graduate Program in Public Health, UNITED STATES

## Abstract

Maternal nutrition during gestation has been investigated for its role in child neurodevelopment. However, little is known about the potential impact of gestational caffeine exposure on child autistic behaviors. Here, we assess the relation between maternal caffeine intake during pregnancy and children’s behavioral traits related to Autism Spectrum Disorder (ASD). We harmonized data from two pregnancy cohorts, Early Autism Risk Longitudinal Investigation (EARLI) (n = 120), an enriched-risk cohort of mothers who previously had a child with ASD, from Pennsylvania, Maryland, and Northern California (2009–2012), and the Health Outcomes and Measures of the Environment (HOME) Study (n = 269), a general population cohort from Cincinnati, Ohio (2003–2006). Mothers self-reported caffeine intake twice during pregnancy. Caregivers reported child behavioral traits related to ASD using the Social Responsiveness Scale (SRS) when children were aged 3–8 years. Higher scores indicate more ASD-related behaviors. We estimated covariate-adjusted differences in continuous SRS T-scores per interquartile range increase in caffeine intake. Self-reported caffeine intake during pregnancy was positively associated with SRS T-scores among children in EARLI (β: 2.0; 95% CI -0.1, 4.0), but to a lesser extent in HOME (β: 0.6; 95% CI -0.5, 1.6). In HOME, pre-pregnancy body mass index (BMI) modified the association between caffeine intake and SRS T-scores, where more positive associations were observed among women with higher BMIs. Our findings suggest gestational caffeine intake may represent a marker of vulnerability to childhood ASD-related behaviors. Additional studies are warranted to extend these findings.

## Introduction

Maternal nutrition during pregnancy has been associated with child health. Specifically, folate, iron, mercury, omega-3 fatty acids, and caffeine have been implicated in the etiology of child neurodevelopment [1–6]. Caffeine is a naturally occurring methylxanthine alkaloid in foods and beverages, and most notable for its stimulation of the central nervous system through its effects on adenosine and GABA receptors [2, 7–9]. The fetus is potentially vulnerable to caffeine given that 75 percent of women in the United States (US) use some form of caffeine during pregnancy, it can cross the placenta and fetal blood brain barrier, and maternal caffeine metabolism decreases during pregnancy [10–13].

The long-term effect of maternal caffeine consumption on neurodevelopment remains inconclusive with most previous epidemiologic studies focusing on outcomes relating to Attention Deficit Hyperactivity Disorder (ADHD) and intelligence (IQ). Several cohort studies have identified associations of gestational caffeine intake with increased child hyperactivity [14, 15] and decreased IQ [16], but not others [17–20]. Findings using rat models identified adverse effects of caffeine intake during gestation on offspring cognition and social behaviors [21–26].

We are unaware of any studies investigating the impact of caffeine on the risk of Autism Spectrum Disorder (ASD), a neurodevelopmental disorder affecting 1.7%– 2.5% percent of US children, characterized by social and communication deficits and restrictive and repetitive stereotypical behaviors, while sharing some features with ADHD [27–31]. Here, we explore the relation between caffeine consumption during pregnancy and ASD-related behaviors in childhood using two prospective cohorts, the Early Autism Risk Longitudinal Investigation (EARLI) and the Health Outcomes and Measures of the Environment (HOME) Study.

## Methods

### Data sources

We used data from the EARLI and HOME studies. EARLI is an enriched-risk prospective pregnancy cohort that recruited pregnant women who already had a child diagnosed with ASD. Details regarding recruitment and data collection have been previously published [32]. Briefly, 264 pregnant women and their biological child with ASD were enrolled (from 806 eligible women) from four sites in the US: Pennsylvania (Drexel/Children’s Hospital of Philadelphia), Maryland (Johns Hopkins/Kennedy Krieger Institute), and Northern California (UC Davis and Northern California Kaiser Permanente) between 2009 and 2012. Biological children with ASD, EARLI study probands, received confirmation of ASD diagnosis by EARLI Study clinicians using the Autism Diagnostic Observation Schedule (ADOS) and either the Mullen Scales of Early Learning or Kaufman Brief Intelligence Test [33–35]. All participating sites’ Institutional Review Boards (IRB) approved the EARLI Study. Eligibility for participation included having a prior child with ASD, being at least 18 years old, less than 29 weeks gestation, communicative in English or Spanish, and living within 2 hours of a study site. All women provided written, informed consent for themselves and their children. From the original sample, 221 women delivered live born, singleton infants. Our analysis included 120 women, after we excluded those without Social Responsiveness Scale (SRS) scores (*n* = 45), caffeine intake data, (*n* = 34), or covariate information (*n* = 22), (S1 Fig).

The HOME Study is a prospective pregnancy and birth cohort study; detailed information regarding recruitment and data collection have been previously published [36]. Briefly, we enrolled 468 pregnant women (1,263 eligible women) between 2003 and 2006 from nine prenatal clinics affiliated with three delivery hospitals in the greater Cincinnati, Ohio area. Inclusion criteria comprised of women being at least 18 years old, 16±3 weeks gestation, HIV-negative, not taking medications for seizures and/or thyroid disorders, and living in homes built before 1978. Women were excluded if they had a diagnosis of diabetes, bipolar disorder, schizophrenia, or cancer the resulted in radiation treatment or chemotherapy. The IRB at Cincinnati Children’s Hospital Medical Center and all participating hospitals approved this study. All participants provided their written, informed consent for themselves and their children. Of 468 women who enrolled, 67 dropped out before delivery. Among the 389 live born, singleton infants, our analysis included 269 mother-child pairs after excluding those with missing data on SRS scores (*n* = 106), caffeine intake, (*n* = 12), and covariate information (*n* = 2) (S1 Fig).

### Data harmonization and pooling

All covariate information was harmonized to accommodate slight differences in study specific information (e.g., categories of income). Both studies used the same outcome measure, the SRS. To pool the data from the two cohorts, we aimed to maximize the inferential equivalence of exposure and covariate measures by harmonizing the data using established procedures [37–42].

### Caffeine exposure assessment

Both studies assessed caffeine intake twice during pregnancy using study-specific questionnaires. Mothers recalled caffeine intake from the 1^st^ half of pregnancy, between conception and through approximately 20 weeks gestation, and for the 2^nd^ half of pregnancy, between approximately 21 weeks gestation and birth.

The caffeine-containing food and beverage items referenced in the dietary recall surveys were similar across the two studies (Supplemental Methods), and included coffee, tea, soda, and chocolate (S1 Table in S1 File). We calculated each subject’s average daily caffeine consumption by considering the type, amount, and frequency of consumption for all caffeine containing foods and beverages. Estimated caffeine intake was based on individual-level daily caffeine consumption. To calculate daily caffeine intake (mg/day) from an item, we multiplied the frequency of consumption of each item per day by the portion size (ounce) and caffeine content of the item (mg/ounce). We then summed the caffeine intake from all items to estimate the daily caffeine intake for each participant. Caffeine content values per serving size were assigned based on United States Department of Agriculture nutrient databases [43]. Caffeine content for specific brand name products were confirmed from product websites. Additional methodological information can be found in the supplemental methods and S1 Table in S1 File.

### ASD-related behavior assessment

ASD-related behaviors were assessed with the SRS, a valid and reliable questionnaire used to measure the presence and severity of ASD-related behaviors in both epidemiological studies and clinical settings [44–50]. The SRS includes 65 items with Likert-scale responses that assess the continuum of traits exhibited by individuals with ASD by measuring interpersonal behaviors, communication, and repetitive/stereotypical behaviors [29, 46]. Item scores are summed and transformed to sex-standardized T-scores (mean:50, standard deviation [SD]:10). Higher scores are indicative of more ASD-related behaviors. Our primary analysis used SRS T-scores. In secondary analyses we used SRS raw and subscale T-scores.

Children’s caregivers completed the SRS when children were ages 3 (*n* = 120) years in EARLI, and 4 (*n* = 189), 5 (*n* = 35), or 8 (*n* = 45) years in HOME. Among HOME Study participants with repeated SRS measures, we used the earliest SRS measurement to be as similar as possible to the measures collected in EARLI. Our prior work showed excellent reproducibility of repeated SRS T-scores in HOME (Intraclass Correlation Coefficient [ICC] = 0.74) [51].

### Covariates

We adjusted for sociodemographic and perinatal factors based on biologic plausibility and *a priori* knowledge using a directed acyclic graph (DAG) (S2 and S3 Figs). Trained research staff administered questionnaires to all participating mothers, collecting information regarding maternal race/ethnicity, age, household income, education, and parity. We assessed smoking during pregnancy using urine (EARLI) and serum (HOME) cotinine concentrations, which were measured by immunoassays and analytic chemistry methods, respectively. When more than one serum cotinine measure was available in HOME, we averaged those values. Cotinine concentrations were used as continuous variables in cohort specific models, and we used established thresholds to determine active versus non-active smoking [52, 53]. In our primary analysis, we adjusted for maternal age, race, parity, cotinine concentrations, and household income.

### Statistical analysis

First, we calculated the central tendency and variation of caffeine intake and child SRS T-scores according to covariates within each cohort. We then calculated ICCs to assess between-and within-person variability in caffeine intake across pregnancy [54]. Our primary analysis used linear regression to estimate the unadjusted and covariate-adjusted difference in continuous SRS T-scores per interquartile range (IQR) increase in continuous caffeine intake based on average, as well as 1^st^ and 2^nd^ half of pregnancy values. This was done for each cohort separately, and pooled. In the pooled data, we examined whether the association between caffeine intake and SRS T-scores varied across the two cohorts using a product interaction term between cohort and caffeine intake. Finally, we used natural cubic splines to examine the shape of the dose-response relations between caffeine intake and SRS T-scores [55].

We conducted several sensitivity analyses. First, we additionally adjusted for child sex and maternal pre-pregnancy BMI. We also considered neonatal intensive care unit (NICU) admission as a potential source of caffeine exposure since high-dose caffeine treatment is often provided to preterm babies to prevent apnea [56]. This information was only available in HOME, and we conducted this analysis by excluding children who were admitted to the NICU. Finally, we adjusted for total energy intake using previously described methods [57]. Total energy intake was only available within EARLI. Finally, we assessed the potential for residual confounders to explain the association between caffeine intake and SRS T-scores, conditional on previously mentioned covariates, we calculated E-values [58].

We conducted several secondary analyses. First, given that previous epidemiological studies found that the association between caffeine intake during pregnancy and childhood neurodevelopmental outcomes varied by source of caffeine, [15, 17, 18, 20] we examined whether the association between caffeine intake and SRS scores varied by caffeine source (coffee, tea, or soda). Second, because ASD is more prevalent in male children than female children [30], we examined whether sex modified the observed associations. We further explored modification by child sex using SRS total raw scores. Third, we assessed the relation between caffeine intake and SRS subscale T-scores (i.e., social awareness, social cognition, social communication, autistic mannerisms, and social motivation). Fourth, using a modified Poisson model, we estimated the relative risk of SRS T-scores ≥60 and ≥75 with increasing caffeine intake. SRS T-scores ranging from 60–75 are indicative of clinically significant deficiencies in reciprocal social behavior that may interfere with daily social interactions, while scores greater than 75 are strongly associated with clinical diagnosis of ASD [59]. Because maternal pre-pregnancy body mass index (BMI) has been identified as a risk factor for atypical child neurodevelopment, including ASD [60–62] we examined whether pre-pregnancy BMI modified the association between caffeine intake and SRS T-scores. Finally, given that smoking is related to both caffeine consumption [63, 64] and an increase in ASD traits in children [65], we conducted additional analyses with and without adjustment for cotinine concentrations in order to assess the strength of this potential confounder. Statistical analyses were completed using R Studio (version 1.1.463) [66].

## Results

Women in the EARLI Study tended to be older and have higher annual incomes than women in the HOME Study (Table 1). Both samples were predominately non-Hispanic White and college-educated. Almost half of HOME Study women were multiparous compared to all of EARLI women (by study design).

**Table 1:** Median maternal self-reported caffeine intake during pregnancy and mean child SRS total T-Scores at 3 to 8 years according to covariates: The EARLI and HOME studies, 2009–2012 and 2003–2006.

Variable			Average ^a^ Caffeine (mg/day)		SRS	
	EARLI	HOME	EARLI	HOME	EARLI	HOME
	N (%)	N (%)	Median (25^th^, 75^th^)	Median (25^th^, 75^th^)	Mean (SD)	Mean (SD)
Overall	120 (100)	269 (100)	20 (8, 65)	18 (5, 48)	52 (13)	51 (10)
Maternal Age						
<25 years	2 (2)	56 (21)	36 (25, 48)	31 (6, 68)	51 (1.4)	57 (12)
25-<35 years	58 (48)	166 (62)	20 (9, 54)	15 (5, 40)	55 (15)	50 (8.1)
35+ years	60 (50)	47 (17)	19 (8, 71)	30 (9, 75)	49 (9.6)	50 (12)
Maternal Race						
White	86 (72)	175 (65)	24 (13, 73)	17 (4, 47)	52 (12)	49 (8.0)
Non-White	34 (28)	94 (35)	12 (5, 32)	24 (9, 49)	53 (14)	56 (12)
Maternal Education						
High School or less	14 (12)	59 (22)	31 (13, 58)	34 (12, 75)	59 (15)	58 (12)
Some College	33 (28)	71 (26)	19 (8, 64)	19 (5, 51)	57 (15)	53 (9.4)
Completed College	73 (60)	139 (52)	20 (8, 67)	15 (4, 38)	49 (9.3)	48 (8.3)
Annual Income						
<$30,000	10 (8)	83 (31)	43 (22, 59)	22 (10, 62)	61 (20)	58 (12)
$30,000–$75,000	38 (32)	83 (31)	19 (8, 77)	15 (4, 42)	55 (14)	51 (9.0)
≥$75,000	72 (60)	103 (38)	19 (8, 50)	16 (5, 46)	50 (9.9)	47 (7.4)
Maternal Smoking ^b^^,^ ^c^						
Non-Smoking	115 (96)	41 (90)	19 (8, 61)	17 (5, 45)	51 (11)	51 (10)
Active Smoking	5 (4)	28 (10)	74 (8, 85)	46 (19, 107)	71 (25)	55 (9.0)
Parity ^d^						
0	N/A	127 (47)	N/A	13 (4, 35)	N/A	51 (10)
1	59 (49)	86 (32)	19 (8, 50)	18 (7, 60)	54 (13)	50 (10)
2	43 (36)	38 (14)	19 (7, 67)	39 (14, 65)	48 (10)	54 (11)
3+	18 (15)	18 (7)	35 (15, 111)	44 (25, 88)	56 (12)	55 (8.9)
Pre-pregnancy BMI (kg/m2) ^e^						
Normal/Underweight <25	48 (40)	136 (53)	17 (8, 40)	15 (5, 48)	49 (11)	51 (10)
Overweight ≥25, <30	30 (25)	66 (25)	20 (14, 53)	19 (6, 43)	51 (11)	51 (8.3)
Obese ≥30	41 (35)	57 (22)	39 (8, 81)	35 (11, 54)	57 (14)	55 (12)
Child Sex						
Male	67 (56)	117 (43)	20 (8, 77)	15 (5, 50)	53 (14)	50 (9.1)
Female	53 (44)	152 (57)	19 (8, 61)	20 (6, 47)	51 (10)	53 (11)

BMI: Body Mass Index, EARLI: Early Autism Risk Longitudinal Investigation Study, HOME: Health Outcomes and Measures of the Environment Study, SRS: Social Responsiveness Scale

^a^ Average self-reported caffeine intake is obtained by calculating the mean estimated daily caffeine intake for all participants from the 1^st^ and 2^nd^ halves of pregnancy.

^b^ Maternal smoking during pregnancy for EARLI was based on maternal urinary cotinine concentrations (a metabolite of nicotine) during pregnancy. The cut off point of 50 ng/ml was used to differentiate between non-smoking and active smoking.[53]

^c^ Maternal smoking during pregnancy for HOME estimated based on maternal serum cotinine concentrations during pregnancy. The cut off point of 3.0 ng/ml was used to differentiate non-smoking and active smoking.[52]

^d^ Note that for parity, the EARLI cohort consists of mothers who had at least one previous child.

^e^ Note that pre-pregnancy BMI information was only available for a subset of each sample (EARLI n = 119; HOME n = 259)

Median daily caffeine intake values were similar in EARLI (20 mg/day) and HOME (18 mg/day) (S4 Fig). Median caffeine intake was inversely related to age in EARLI, but we observed a ‘U’ shaped pattern in HOME. Caffeine intake was highest in White women in EARLI, and non-White women in HOME. In both cohorts, caffeine intake was positively related to smoking and parity, and inversely related to maternal education and household income. The reproducibility of caffeine intake between 1^st^ and 2^nd^ half of pregnancy was good in EARLI (ICC = 0.6, 95% CI: 0.5, 0.7) and fair in HOME (ICC = 0.4, 95% CI: 0.2, 0.5) [54]. The distributions of child SRS T-scores were similar in EARLI (mean: 52, SD: 13) and HOME (mean: 51, SD: 10). While the proportion of children with SRS scores ≥ 60 were the same EARLI (n = 20, 17%) and HOME (n = 43, 16%), the proportion of children with SRS scores ≥75 was higher in EARLI (n = 10, 8%) compared to HOME (n = 11, 4%) (S5 Fig). In both cohorts, SRS T-scores were inversely associated with maternal age, education, and, income, and positively associated with smoking and pre-pregnancy BMI.

### Maternal caffeine intake and social responsiveness scores

After adjustment for maternal age, race, income, parity, and smoking status, we observed a positive association between caffeine intake and SRS T-scores in EARLI (β per IQR increase [57mg]: 2.0, 95% CI: -0.1, 4.0), and to a lesser extent in HOME (β per IQR increase [43mg]: 0.6, 95% CI: -0.5, 1.6) (Table 2). When considering caffeine intake as a continuous measure, a 1-mg increase in caffeine intake is associated with an increase in SRS T-scores equivalent to (β: 0.03, 95% CI: 0.00, 0.07) in EARLI, and (β: 0.01, 95% CI: -0.01, 0.04) in HOME (S2 Table in S1 File). Within EARLI, the association was slightly stronger for caffeine intake in the 2^nd^ half of pregnancy (*β* per IQR increase: 1.8, 95% CI: 0.4, 3.2) than the 1^st^ (*β* per IQR increase: 1.0, 95% CI: -1.0, 3.0). The association between caffeine intake and SRS T-scores did not differ substantially between the 1^st^ and. 2^nd^ half of pregnancy within HOME (Table 2). In the pooled sample, average caffeine intake was positively associated with SRS T-scores (*β* per IQR increase: 1.2, 95% CI: 0.3, 2.1), although the cohort x caffeine interaction term indicated significant heterogeneity across the cohorts (*P* value = 0.02) (S3 Table in S1 File).

**Table 2:** Unadjusted and adjusted differences in children’s SRS T-score at ages 3 to 8 per IQR increase in maternal self-reported caffeine intake during pregnancy: The EARLI and HOME studies 2009–2012 and 2003–2006 ^a^^,^ ^b^.

Cohort/Gestational Period	IQR (range) ^c^	Unadjusted	Adjusted
		β (95% CI)	β (95% CI)
EARLI (n = 120)			
Average	57 (8, 65)	2.5 (0.4, 4.6)	2.0 (-0.1, 4.0)
1^st^ Half (<20 weeks)	66 (6, 72)	1.8 (-0.2, 3.9)	1.0 (-1.0, 3.0)
2^nd^ Half (>20 weeks)	43 (0, 43)	1.9 (0.4, 3.3)	1.8 (0.4, 3.2)
HOME (n = 269)			
Average	43 (5, 48)	1.0 (0.0, 2.1)	0.6 (-0.5, 1.6)
1^st^ Half (<20 weeks)	46 (1, 47)	0.8 (0.0, 1.7)	0.4 (-0.5, 1.3)
2^nd^ Half (>20 weeks)	38 (4, 42)	0.5 (-0.4, 1.3)	0.3 (-0.5, 1.1)

EARLI: Early Autism Risk Longitudinal Investigation Study, HOME: Health Outcomes and Measures of the Environment Study, IQR: Interquartile Range, SRS: Social Responsiveness Scale

^a^ Adjusted for maternal age (continuous), maternal race (white vs non-white), income (<$30,000 vs $30,000-$75,000, ≥ $75,000), parity (continuous), and log10 –transformed urine/serum cotinine concentrations (continuous). Note cotinine concentrations were ascertained from maternal urine in EARLI and serum in HOME.

^b^ Positive coefficients for SRS indicate that maternal caffeine intake is associated with more deficits in social responsiveness traits.

^c^ IQR values listed in mg caffeine /day

**Fig 1:**
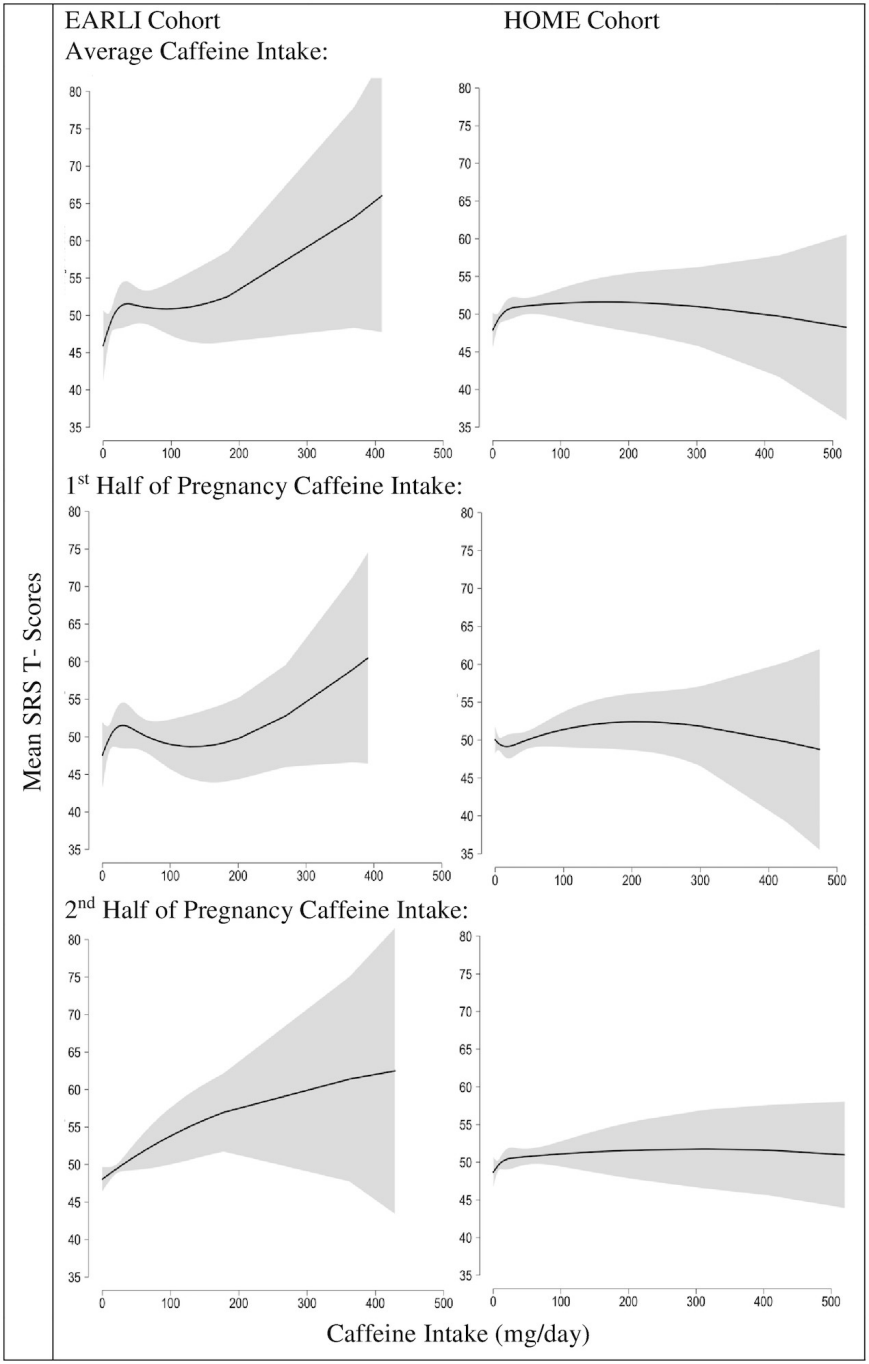
Adjusted mean child SRS total T-scores at ages three to eight by maternal self-reported caffeine intake during pregnancy, derived from a natural spline: The EARLI and HOME studies. SRS: Social Responsiveness Scale, EARLI: Early Autism Longitudinal Investigation, HOME: Health Outcomes and Measures of the Environment. Adjusted for maternal age (continuous), maternal race (white vs non-white), income (<$30,000 vs $30,000-$75,000, ≥$75,000), parity (continuous), and log10 –transformed urine/serum cotinine concentrations (continuous). Note cotinine concentrations were ascertained from maternal urine in EARLI and serum in HOME. Bands are the 95% confidence intervals.

Using natural splines to examine the dose-response relation, we observed a relatively monotonic positive association of caffeine intake with SRS T-scores in EARLI (Fig 1). In HOME, SRS T-scores increased with maternal caffeine intake up to 100 mg/day, but plateaued at levels greater than 100mg/day. In both studies, the 95% CIs were imprecise at higher caffeine intake values. Within the pooled cohort, we observed modest, positive associations between caffeine intake and SRS T-scores (S6 Fig).

### Sensitivity and secondary analyses

Adjusting for child sex, pre-pregnancy BMI, total energy intake, and NICU admission did not substantially change our results (S4 Table in S1 File). When examining the association between caffeine intake and SRS T-scores by caffeine source, caffeine intake from tea showed the largest positive association with SRS scores compared to coffee and soda in both cohorts (S5 Table in S1 File). Caffeine intake from coffee and tea was higher in EARLI compared to HOME where caffeine intake was highest for soda (S6 Table in S1 File). However, the 95% CIs of the associations for the caffeine from tea were imprecise and overlapped the point estimates of associations between other sources of caffeine and SRS T-scores. The association between average caffeine intake and SRS T-scores did not vary by child sex in either cohort. When using SRS total raw scores, which are not standardized for child sex, the patterns of associations remained similar (S7 Table in S1 File). Moreover, the patterns of association of caffeine intake with SRS T-score subscales were also similar to SRS Total T-scores (S8 Table in S1 File).

Within EARLI and HOME, 20 (17%) and 43 (16%) children had SRS T-scores ≥60, while 10 (8%) and 11 (4%) children had SRS T-scores ≥75, respectively. In both cohorts, the relative risks of having SRS T-scores ≥60 with increasing average caffeine intake during pregnancy was <1.1 in both cohorts (S9 Table in S1 File). However, there was a modest, but imprecise, increase in the risk of having SRS T-scores ≥75 with increasing average caffeine intake in both EARLI (RR per IQR increase: 1.3, 95% CI: 0.8, 2.3) and HOME (RR per IQR increase: 1.4, 95% CI: 0.9, 2.4).

In adjusted models, the strength of caffeine-SRS T-score associations in EARLI increased when not adjusting for maternal cotinine; however, there were no substantial differences in HOME. It should be noted that few participants in EARLI (*n* = 5) were identified as active smokers based on urine cotinine concentrations (> 50 ng/mL) [53] (S10 Table in S1 File).

**Fig 2:**
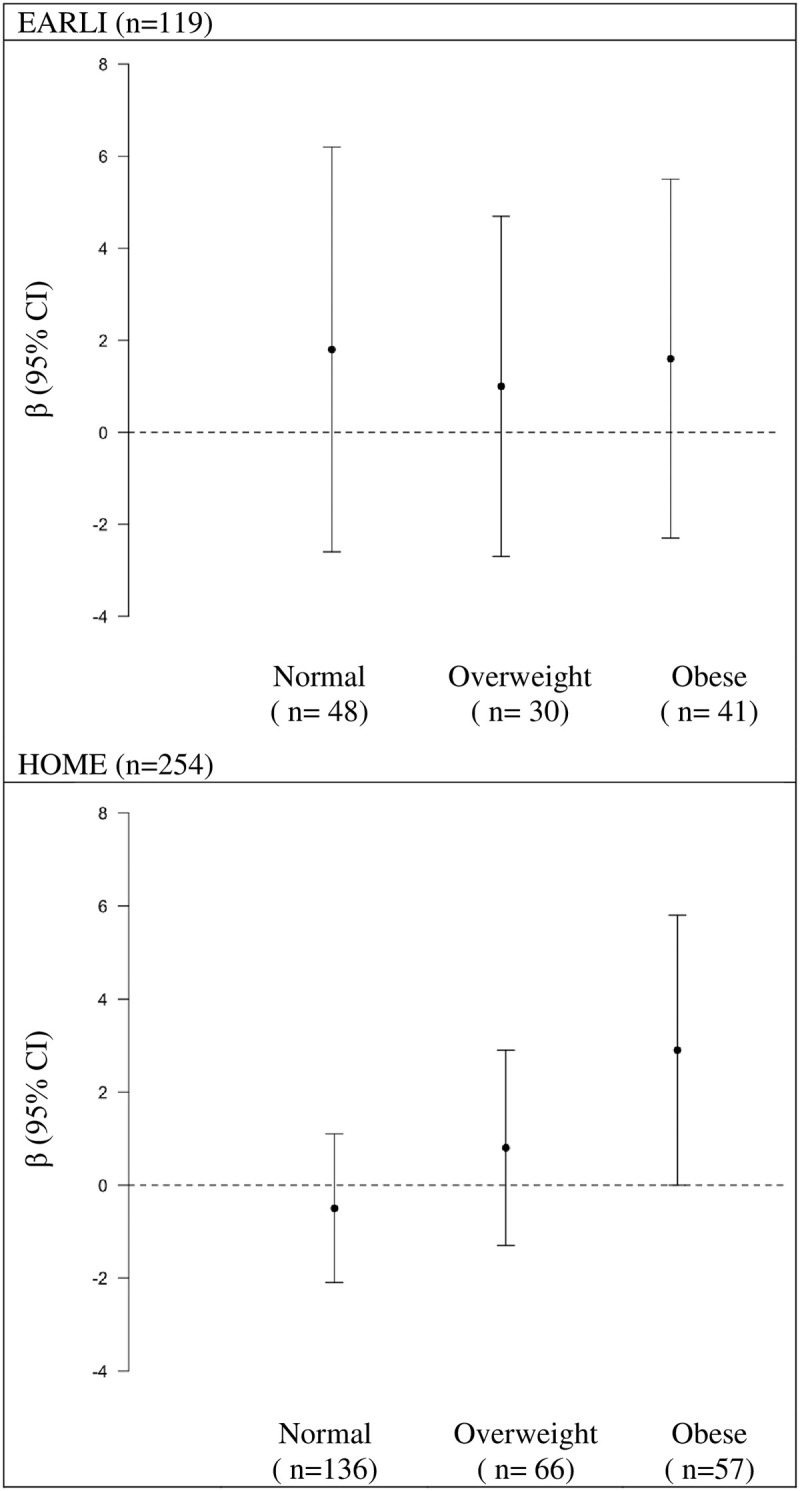
Adjusted differences in children’s SRS T-score at ages three to eight per IQR increase in self-reported caffeine intake during pregnancy stratified by maternal pre-pregnancy BMI category: The EARLI and HOME studies. SRS: Social Responsiveness Scale, EARLI: Early Autism Longitudinal Investigation, HOME: Health Outcomes and Measures of the Environment, IQR: Interquartile Range, BMI: Body mass index. Adjusted for maternal age (continuous), maternal race (white vs non-white), income (<$30,000 vs $30,000-$75,000, ≥$75,000), parity (continuous), and log10 –transformed urine/serum cotinine concentrations (continuous). Note cotinine concentrations were ascertained from maternal urine in EARLI and serum in HOME. Positive coefficients for SRS indicate that maternal caffeine intake is associated with more deficits in social responsiveness traits. Normal/Underweight: BMI <25, Overweight: BMI ≥25<30, and Obese BMI ≥30.

When examining whether maternal pre-pregnancy BMI modified the association between caffeine intake and SRS T-scores, we categorized women as obese (BMI: ≥30 kg/m^2^), overweight (BMI: 25-<30 kg/m^2^), and normal weight (BMI: 18.5<25 kg/m^2^) or underweight (BMI: <18.5 kg/m^2^) [67]. In HOME, but not EARLI, we observed that pre-pregnancy BMI modified the association between caffeine intake and SRS T-scores (BMI category x caffeine interaction *P* value = 0.04) (S11 Table in S1 File). In HOME, the positive relation between caffeine intake and SRS T-scores was stronger with increasing maternal pre-pregnancy BMI (Fig 2).

## Discussion

We observed a modest positive association between maternal self-reported caffeine intake during pregnancy and SRS T-scores in children. These associations were stronger in EARLI, the ASD enriched-risk cohort, than in HOME, the general population cohort and, robust to adjustment for child sex, pre-pregnancy BMI, total energy intake, and NICU admission. Additionally, we observed that pre-pregnancy BMI modified the association between maternal caffeine intake and SRS T-scores in HOME. Recognizing the differences between these cohorts in terms of recruitment and study sample characteristics, the stronger, positive association between caffeine intake during pregnancy and SRS T-scores in children in EARLI relative to HOME may reflect the differences in underlying ASD susceptibility that exists within these study populations.

To our knowledge, the association between gestational caffeine intake and children’s autistic behaviors has not been previously investigated. Thus, we place these findings in the context of prior studies evaluating associations between maternal caffeine intake and child neurodevelopment more broadly given the frequency of co-occurring psychiatric conditions with ASD [29, 31, 68]. For example, previous reports have investigated the association between maternal caffeine intake during gestation with child hyperactivity and cognition. One cohort study reported no association between maternal caffeine intake from tea and coffee specifically with clinically diagnosed ADHD in children [18]. However, two other cohort studies found that caffeine intake from soda, but not all source caffeine intake, was associated with child hyperactivity [14, 15]. Here, we did not observe strong evidence to suggest caffeine source influences child SRS T-scores. Another cohort study found that caffeine consumption over 200 mg/day was associated with decreases in child IQ [16], although others report null findings [17, 19]. Overall, most previous studies observe a monotonic dose-response relationship between maternal caffeine intake and child neurodevelopmental outcomes [16, 20]. Differences in the results of the present study and prior ones could be due to the neurodevelopmental outcomes assessed or range of maternal caffeine intake values. Prior studies evaluated maternal caffeine intake at least two times during pregnancy [14–16], and reported median caffeine intake levels between 40 and 200 mg/day [14–18, 20] which are higher than EARLI (18 mg/day) and HOME (20 mg/day). In addition, median caffeine intake in the EARLI and HOME Studies are *lower than* caffeine intake values among pregnant women participating in contemporary cohort studies (medians: 50–60 mg/day) [69, 70]. Given the lower range of caffeine intake in EARLI and HOME, then this would limit our power to detect associations with child SRS T-scores.

A limitation among epidemiologic studies evaluating maternal caffeine intake and child neurodevelopment is the reliance on food frequency questionnaires and dietary recall surveys to estimate daily caffeine intake. While having multiple measures of maternal caffeine intake in this and prior studies may reduce measurement error relative to studies which only evaluated maternal caffeine intake at one time point throughout the pregnancy period [20], differences in how caffeine intake was estimated across studies could contribute to discrepant findings. For example, studies considering caffeine intake from multiple food and beverage items [14, 16, 69, 70] may be less biased and more likely to capture true levels of daily maternal caffeine intake than studies which only considered caffeine intake from coffee and tea [15, 18, 20].

Results from animal literature generally support a positive association between increased maternal caffeine intake with more atypical offspring neurodevelopmental traits. These include increased offspring hyperactivity [24, 26, 71, 72], atypical social behaviors [25, 26], as well as anxiety and depressive behaviors [21, 73]. Increased caffeine consumption has also been associated with deficits in offspring learning capabilities and cognitive function [21–24]. However, in experimental studies, pure caffeine was used as the chemical exposure, and levels of caffeine intake are much higher (e.g., equivalent to as much as 12 cups of coffee per day) [23, 25, 71, 73] than those in this and prior studies. This suggests that the neurotoxic effects of caffeine may only be present at high levels of caffeine intake, and thus given the low range of caffeine intake levels reported amongst women in the EARLI and HOME studies, it is plausible that caffeine intake was not high enough to be associated with neurodevelopment.

We speculate that differences in the magnitude of the associations in the EARLI and HOME studies could be due to differences between genetic loading and how this may modify the impact of environmental risk factors. Given that caffeine influences GABA receptors [2], and that GABAergic pathways have been implicated in the etiology of ASD [71], the larger caffeine-SRS association in EARLI (ASD-enriched risk cohort) compared to HOME could be due to increased genetic loading (e.g. alterations in GABA related genes). This could then increase the susceptibility of the fetus to environmental risk factors acting on shared biological pathways.

Our study has several strengths and limitations that should be considered when interpreting these findings. First, both cohorts were prospective in design and included measures of caffeine intake collected at two time points during pregnancy, allowing us to reduce within-person variation in caffeine intake by averaging the two measures. While averaging multiple measures of caffeine intake can reduce within-person variation, it is possible that women may change their dietary patterns during pregnancy [72, 74–76]. Future work could consider identifying specific periods of susceptibility to caffeine intake in relation to autistic behaviors, as prior studies of folic acid and ASD risk suggest that the periconceptional period may be especially important [3]. Moreover, caffeine intake was determined by considering multiple sources of caffeine, allowing us to obtain a more comprehensive measure of exposure. However, the food frequency questionnaires utilized in both the EARLI and HOME Studies were not intentionally designed to capture caffeine intake and several factors discussed below may contribute to measurement error and the relatively low levels of estimated caffeine intake in both cohorts. In EARLI we were unable to account for caffeine intake from espresso and coffee drinks (e.g., lattes, mochas, etc.). Thus, we estimated caffeine content from ‘caffeinated coffee’ using national data on consumption of different types of coffee beverages to obtain a weighted average of an 8 oz cup of coffee [77]. Moreover, we were able to ascertain more precision in the types of caffeine containing food and beverage items assessed in HOME compared to EARLI, thus it is possible caffeine intake values were less biased. Finally, while dietary recalls are subject to some bias, caffeine intake measures (Caffeine Consumption Questionnaire [78], and structured dietary recall interviews) have been validated against salivary caffeine and urinary caffeine metabolite concentrations [73–80] Future work would benefit from investigating the association between child neurobehavior and caffeine intake assessed via biomarkers or more specific questionnaires.

Relatedly, BMI, an effect measure modifier identified here, may be related to differential misreporting of dietary habits, although how this affects caffeine intake is unclear [74]. However, it is possible that women who are overweight and obese may also have higher soda and sugar sweetened beverage consumption, and may also be more likely to underreport their consumption. This could lead to differential bias if being overweight or obese (or factors related to it) are associated with SRS scores [81].

Harmonizing daily caffeine intake relied on several assumptions. For most of these sources of error in caffeine measurement, we expect that any such caffeine measurement error biases are likely to be non-differential with respect to SRS T-scores. However, if the pattern of caffeine intake reporting differs by cohort based on having a previous child with ASD (i.e., EARLI cohort), women are more likely to report SRS scores and caffeine intake differently than women in HOME, then there could be differential misclassification. Additionally, the individual and combined use of harmonizing the enriched-risk ASD cohort (EARLI) and the general population cohort (HOME) is a unique opportunity to attempt replication in two cohorts, and thus is a strength of this work. The comparison of results between these two cohorts, provides an opportunity to discern if environmental exposures (maternal caffeine intake during pregnancy) differentially affects those who have more genetic risk factors for ASD compared to the general population.

While we were able to examine the association between maternal caffeine intake and ASD-related behaviors in two cohorts, differences in features of these participants from these cohorts may be partly responsible for the heterogeneous results we observed with respect to cohort. In addition, there were features unique to each cohort that could not be accounted for, including multiparity as an eligibility requirement in EARLI. Further, both studies used different dietary recall surveys to account for caffeine intake during pregnancy. Differences in the questions asked, and precision available to more accurately estimate daily maternal caffeine intake could have also contributed to the differences in findings between the cohorts.

Third, while the SRS provides a continuous measure of autistic behaviors and traits, it is not a clinical diagnosis of ASD [44]. However, SRS T-scores have been related to the same genetic factors and neuroimaging features as ASD diagnosis, suggesting it validly measures key traits central to the condition [75, 76]. It should also be noted that while the mean SRS T-score values were similar for both EARLI and HOME, the proportion of children with SRS T-scores ≥60, indicative of clinically significant deficiencies associated with clinical diagnosis of ASD [59], was higher in EARLI compared to HOME. This was expected given then ASD-enriched nature of the EARLI cohort.

Fourth, while we adjusted for many potential confounders, our results suggest that residual confounding could be present, especially when examining relatively modest effect sizes. For example, dietary patterns, nausea, or caffeine aversion during pregnancy could alter maternal dietary patterns and food choices [74–76]. Within EARLI, the association between caffeine and SRS scores were attenuated after adjustment for maternal cotinine concentrations, suggesting that behavioral and lifestyle factors are responsible for differences between unadjusted and adjusted estimates. In HOME, the differences between the crude and adjusted estimates were driven primarily by maternal race and income. Based on an E-value calculation, the observed associations could be explained by an unmeasured confounder that was associated with both maternal caffeine intake and child SRS-T scores with a β value (and lower 95% CI) of 1.6 (1.0), 1.3 (1.0), and 1.4 (1.2) for EARLI, HOME, and the pooled cohort, respectively, above and beyond the measured confounders [58, 82, 83]. However, weaker confounding could not do so. Finally, we were unable to consider some potential confounders (e.g. NICU admission) in both cohorts.

Given the differences in the recruitment of participants for each of the studies considered here, our findings may be limited in their generalizability. Both samples were predominately White and college educated. However, we had the opportunity to assess the presented association in two demographically similar cohorts. In order to evaluate potential selection bias, we did assess the sociodemographic factors of those with missing covariate information and/or caffeine intake data; and generally there were no substantial differences based on participants excluded for missing data. In EARLI, sociodemographic factors were similar among those missing covariates and those included in the analysis, with the exception of child sex. Those missing caffeine data were more likely to have lower income than the study sample. In the HOME Study, women missing caffeine data were more likely to have lower household income and education than those included in the analysis. Selection bias is a potential limitation if these factors are related to both caffeine intake and SRS scores.

## Conclusion

In EARLI, and to a lesser extent in HOME, higher maternal caffeine intake during pregnancy was associated with modest increases in ASD-related behaviors among 3–8 year old children. We speculate that differences in the magnitude of these associations across the two cohorts could be due to the individual and combined influences of genetic and environmental susceptibilities that underlie ASD-related traits. Comparing findings from both cohorts could identify potentially modifiable markers of ASD-susceptibility within those with more genetic risk factors comparted to the general population. Future work should examine the potential role of caffeine in ASD etiology using larger samples, more reliable and valid measures of caffeine intake, and adjusting for behavioral and lifestyle factors associated with caffeine intake during pregnancy.

## Supporting information

S1 File (DOCX)

S1 Fig Flow chart of participant selection to final sample size. (TIF)

S2 Fig Directed acyclic graph used to select covariates for our primary analysis in the association between maternal self-reported caffeine intake and child SRS scores. (TIF)

S3 Fig Directed acyclic graph used to select covariates for our primary and secondary analyses in the association between maternal self-reported caffeine intake and child SRS score. (TIF)

S4 Fig Violin plot of maternal self-reported caffeine intake during the 1^st^ and 2^nd^ half of pregnancy: The EARLI and HOME studies, 2009–2012 and 2003–2006. ^a^ Median values of self-reported caffeine intake for each time period have been marked with a dark circle. Median values for maternal self-reported caffeine intake for EARLI: Average: 20mg/day, 1^st^ half of pregnancy: 28mg/day, 2^nd^ half of pregnancy: 7 mg/day; HOME: Average: 18 mg/day, 1^st^ half of pregnancy:17mg/day, 2^nd^ half of pregnancy: 15mg/day ^b^ Average caffeine intake values were estimated from the 1^st^ and 2^nd^ halves of pregnancy exposure measures. ^c^ Each graph shown is a density function, and represents the distribution of maternal self-reported caffeine intake at each time point.
(TIF)

S5 Fig Kernel density plot of distributions of Children’s SRS T-Score at Ages 3: the EARLI and HOME studies, 2009–2012 and 2003–2006. ^a^ The central tendencies of child SRS T-scores were similar in EARLI (mean: 52, SD: 13) and HOME (mean: 51, SD: 10). ^b^ SRS T-scores ranging from 60–75 are indicative of clinically significant deficiencies in reciprocal social behavior that may interfere with daily social interactions, while scores greater than 75 are strongly associated with clinical diagnosis of ASD.
(TIF)

S6 Fig Adjusted mean children’s SRS T-score at ages 3 by maternal self-reported caffeine intake during pregnancy, derived from a natural spline: the EARLI and HOME studies, 2009–2012 and 2003–2006. ^a^ Adjusted for maternal age (continuous), maternal race (white vs non-white), income (<$30,000 vs $30,000-$75,000, ≥$75,000), parity (continuous), smoking during pregnancy as a binary variable, and cohort. Log10 –transformed urine/serum cotinine concentrations (continuous) were used to determine smoking status. Note cotinine concentrations were ascertained from maternal urine in EARLI and serum in HOME.
(TIF)
