# Supplementary material for: Association between self-reported caffeine intake during pregnancy and social responsiveness scores in childhood: The EARLI and HOME studies
Source: PLoS One. 2021 Jan 15;16(1):e0245079. doi: 10.1371/journal.pone.0245079 (PMC7810310; doi:10.1371/journal.pone.0245079)
Supplement: S1 File — (DOCX) [file pone.0245079.s001.docx]

**Supplemental methods**

**Harmonization of caffeine intake assumptions**

Self-reported caffeine intake during pregnancy was assessed using cohort-specific survey questionnaires in the EARLI and HOME Studies. We attempted to maximize the inferential equivalence of caffeine intake variables across the two cohorts with respect to the amount, frequency, and type of caffeine-containing food or beverage consumption.

We considered the relative frequency of consumption for each type of caffeine containing beverage or food. We then multiplied the frequency of consumption of each beverage or food by the portion size, and multiplied this by the caffeine content of the respective product. We used estimates of beverage or food-specific caffeine content from the United States Department of Agriculture’s Agricultural Research Service Food Composition Database, the National Coffee Association, and the Tea Council of the USA.^43,81,82^ In assigning caffeine content for all sources considered, we assumed all items contained caffeine unless specified as caffeine-free. For reference, caffeine content in 8 fluid ounces of hot coffee, tea, or chocolate were assigned values of 95, 29, and 5 mg, respectively. For caffeinated soft drinks, we assumed a serving size of 12 fluid ounces, and assigned 33 mg of caffeine per serving. Chocolate candy was assigned 43 mg of caffeine based on a 1.5 ounce size candy bar.

In cases where the response on a question provided a range of values for frequency or portion size (e.g., 1-2 times per day), we used the average of that range (e.g. 1.5 times per day). When consumption of beverages was determined based on ‘cups’ or ‘bottles,’ we assumed that serving size referred to the standard 8 fluid ounce cup or 12 fluid ounce bottle. Unless a specific brand name was mentioned in the questionnaire, we assigned caffeine values based on the most generic food item within the USDA database. When specific brand names were mentioned, we confirmed caffeine content based on the product’s official company website. In cases where participants could write in their own response, caffeine values were assigned individually, and grouped accordingly based on the source of caffeine.

When a participant could not recall how often they typically consumed a particular beverage or food item, we assigned them an intake value of 0. It is important to note however, that this was rare in both cohorts, occurring in 3 (2.5 percent) EARLI and 1 (0.4 percent) HOME participant. If a participant endorsed consumption of a specific food item, but did not identify the portion size, we assigned them the average value. For responses including a ‘greater than’ option, we assigned the largest value option to participants endorsing that category. If the participant endorsed ‘almost never’, we assigned a value of 0.

**Caffeine exposure assessment assumptions**

The cohort-specific different food frequency questionnaires used in each study allowed us to calculate varying levels of resolution regarding caffeine intake for each cohort. For example, in HOME, we were able to calculate that an individual who drank instant coffee consumed less caffeine than someone who drank drip coffee (See table S1). However, this level of resolution was not captured under the item 'caffeinated coffee' in the EARLI Study. In order to obtain a more accurate caffeine content for 'caffeinated coffee', for EARLI participants, we obtained distribution types of different types of coffee beverages intake within the US in order to weight what an 'average' cup of caffeinated coffee would be.^82^ This measure included the types of coffee beverages similarly asked about in the HOME questionnaire, such as instant coffee, drip coffee, and espresso drinks. This similar method was used to obtain a weighted caffeine content value for tea.^81^

**Sections from dietary recall surveys**

**Caffeine specific items from participant dietary recall survey: The EARLI Study**

During the first (or later) 20 weeks of your current pregnancy…

1. How often did you drink soft drinks, soda, or pop?

- 1 time per month
- 2-3 times per moth
- 1-2 times per week
- 3-4 times per week
- 5-6 times per week
- 1 time per day
- 2-3 times per day
- 4-5 times per day
- 6 or more times per day
- Don’t know
- Never

1a. Each time you drank soft drinks, soda, or pop, how much did you usually drink?

- Less than 12 ounces or less than 1 can or bottle
- 12-16 ounces of 1 can or bottle
- More than 16 ounces or more than 1 can or bottle
- Don’t know

1b. How often were these soft drinks, soda, or pop diet or sugar-free?

- Almost never or never
- About ¼ of the time
- About ½ of the time
- About ¾ of the time
- Almost always or always
- Don’t know

1c. How often were these soft drinks, soda, or pop caffeine-free?

- Almost never or never
- About ¼ of the time
- About ½ of the time
- About ¾ of the time
- Almost always or always
- Don’t know

2. How often did you each chocolate candy?

- 1 time per month
- 2-3 times per moth
- 1 time per week
- 2 times per week
- 3-4 times per week
- 5-6 time per week
- 1 time per day
- 2 or more times per day
- Don’t know
- Never

2a. Each time you ate chocolate candy, how much did you usually eat?

- Less than 1 average bar or less than 1 ounce
- 1 average bar or 1 to 2 ounces
- More than 1 average bar or more than 2 ounces
- Don’t know

3. How many cups of coffee, caffeinated or decaffeinated did you drink?

- 1-3 cups per month
- 1 cup per week
- 2-4 cups per week
- 5-6 cups per week
- 1 cup per day
- 2-3 cups per day
- 4-5 cups per day
- 6 or more cups per day
- Don’t know
- Never

3a. How often was the coffee you drank decaffeinated?

- Almost never or never
- About ¼ of the time
- About ½ of the time
- About ¾ of the time
- Almost always or always
- Don’t know

4. How many glasses of iced tea, caffeinated or decaffeinated, did you drink?

- 1-3 cups per month
- 1 cup per week
- 2-4 cups per week
- 5-6 cups per week
- 1 cup per day
- 2-3 cups per day
- 4-5 cups per day
- 6 or more cups per day
- Don’t know
- Never

4a. How often was the iced tea you drank decaffeinated or herbal tea?

- Almost never or never
- About ¼ of the time
- About ½ of the time
- About ¾ of the time
- Almost always or always
- Don’t know

4b. How often was the iced tea you drank green tea?

- Almost never or never
- About ¼ of the time
- About ½ of the time
- About ¾ of the time
- Almost always or always
- Don’t know

5. How many cups of hot tea, caffeinated, decaffeinated, or herbal did you drink?

- 1-3 cups per month
- 1 cup per week
- 2-4 cups per week
- 5-6 cups per week
- 1 cup per day
- 2-3 cups per day
- 4-5 cups per day
- 6 or more cups per day
- Don’t know
- Never

5a. How often was the hot tea you drank decaffeinated or herbal tea?

- Almost never or never
- About ¼ of the time
- About ½ of the time
- About ¾ of the time
- Almost always or always
- Don’t know

4b. How often was the hot tea you drank green tea?

- Almost never or never
- About ¼ of the time
- About ½ of the time
- About ¾ of the time
- Almost always or always
- Don’t know

**Caffeine specific items from participant dietary recall survey: The HOME Study**

1. Did you drink any of the following types of coffee in the last month (check all that apply)

- Instant (caffeinated) coffee
- Percolated (caffeinated) coffee
- Drip (caffeinated) coffee
- Expresso
- Decaffeinated coffee
- Others (please specify)
- None of the above
- Refuse
- Don’t know

1a. On average, how many cups of {complete for each type of coffee previously indicated} per day did you drink in last month (cups of coffee should be in multiples of 8oz except for expresso which is 4ozs, if not drinking 8oz cups (4 oz cups for espresso) ask for ounces then convert to cups using 8oz=1 cup (for coffee), 4 oz = 1 cup (for expresso)

- ______ per day
- ______ per week (if less than 1 per day)
- ______ per month (if less than 1 per week)
- Refuse
- Don’t know

2. Did you drink any of the following tea (iced or hot) in last month? (check all that apply)

- Any decaffeinated tea
- Any herbal tea
- Instant (caffeinated tea)
- Caffeinated tea made from using tea bags
- Others (please specify)
- Refuse
- Don’t know
- None of the above

2a. On average, how many cups of {complete for each type of tea previously indicated} per day did you drink in last month (cups of tea should be in multiples of 8oz, if not drinking 8oz cups ask for ounces then convert to cups using 8oz=1 cup)

- ______ per day
- ______ per week (if less than 1 per day)
- ______ per month (if less than 1 per week)
- Refuse
- Don’t know

3. On average, how many cups of hot chocolate per day did you drink in last month? (cups of hot chocolate should be in multiples of 8 oz, ask for ounces if not drinking 8-oz cups then convert to cups using: 8ozs = 1 cups)

- ______ per day
- ______ per week (if less than 1 per day)
- ______ per month (if less than 1 per week)
- Refuse
- Don’t know

4. Did you drink any of the following soft drinks in last month? (check all that apply)

- Planet Java Tremble
- Planet Java Caramocha or Javadelic
- Pepsi One, Mountain Dew, Mellow Yellow, Surge, Kick
- Pepsi-Cola (all except Pepsi One), Coca Cola (all except diet Coke), Ruby Red, Storm, Big Red, Snapple flavored teas, KMX
- Diet coke, Dr. Pepper, Tab, Shasta Cola, Dr Nehi, Mr Pibb, Red Flash, Squirt, Sun Drop, Sunkist
- Canada Dry Cola, A&M, Crème Soda, Nestea Iced Tea, Barqs root beer
- Mistic Tea, Lipton Brisk Tea
- Refuse
- Don’t know
- None of the above

4a. On average, how many cans of soft drinks like {complete for each type of soft drink previously indicated} per day did you drink in last month? (cans of soft drinks should be in multiples of 12ozs, ask for ounces if not drinking 12 oz cans then convert to cans using: 12 oz = 1 can)

- ______ per day
- ______ per week (if less than 1 per day)
- ______ per month (if less than 1 per week)
- Refuse
- Don’t know

5. Did you drink any other soft drinks in last month? (enter names of soft drinks below)

- ______ Name 1
- ______ Name 2
- ______ Name 3
- ______ Name 4
- ______ Name 5
- ______ Name 6
- ______ Name 7
- ______ Name 8
- No other soft drinks drunk
- Refuse
- Don’t know

5a. On average, how many cans of {complete for each type of other soft drink previously indicated} per day did you drink in last month? (cans of soft drinks should be in multiples of 12ozs, ask for ounces if not drinking 12 oz cans then convert to cans using: 12 oz = 1 can)

- ______ per day
- ______ per week (if less than 1 per day)
- ______ per month (if less than 1 per week)
- Refuse
- Don’t know

**S1 Figure. Flow Chart of Participant Selection to Final Sample Size.**

**S2 Figure. Directed Acyclic Graph used to Select Covariates for our Primary Analysis in the Association Between Maternal Self-Reported Caffeine Intake and Child SRS Scores.**

**S3 Figure. Directed Acyclic Graph used to Select Covariates for our Primary and Secondary Analyses in the Association Between Maternal self-reported Caffeine Intake and Child SRS Score.**

**S4 Figure. Violin Plot of Maternal Self-Reported Caffeine Intake During the 1^st^ and 2^nd^ Half of Pregnancy: the EARLI and HOME Studies, 2009-2012 and 2003-2006.** ^a^ Median values of self-reported caffeine intake for each time period have been marked with a dark circle. Median values for maternal self-reported caffeine intake for EARLI: Average: 20mg/day, 1^st^ half of pregnancy: 28mg/day , 2^nd^ half of pregnancy: 7 mg/day; HOME: Average: 18 mg/day, 1^st^ half of pregnancy:17mg/day , 2^nd^ half of pregnancy: 15mg/day. ^b^ Average caffeine intake values were estimated from the 1^st^ and 2^nd^ halves of pregnancy exposure measures. ^c^ Each graph shown is a density function, and represents the distribution of maternal self-reported caffeine intake at each time point.

**S5 Figure. Kernel density plot of distributions of Children’s SRS T-Score at Ages 3: the EARLI and HOME Studies, 2009-2012 and 2003-2006.** ^a^ The central tendencies of child SRS T-scores were similar in EARLI (mean: 52, SD: 13) and HOME (mean: 51, SD: 10). ^b^ SRS T-scores ranging from 60-75 are indicative of clinically significant deficiencies in reciprocal social behavior that may interfere with daily social interactions, while scores greater than 75 are strongly associated with clinical diagnosis of ASD.

**S6 Figure. Adjusted mean Children’s SRS T-Score at Ages 3 by maternal self-reported caffeine intake during pregnancy, derived from a natural spline: the EARLI and HOME Studies, 2009-2012 and 2003-2006.** ^a^ Adjusted for maternal age (continuous), maternal race (white vs non-white), income (<$30,000 vs $30,000-$75,000, ≥$75,000), parity (continuous), smoking during pregnancy as a binary variable, and cohort. Log_10_ –transformed urine/serum cotinine concentrations (continuous) were used to determine smoking status. Note cotinine concentrations were ascertained from maternal urine in EARLI and serum in HOME.

| S1 Table. Caffeine Content Assigned to All Food and Beverage Items Referenced Within Maternal Self-Reported Dietary Recall Questionnaires Administered in the 1^st^ and 2^nd^ Halves of Pregnancy: The EARLI and HOME Studies, 2009-2012 and 2003-2006. | | | | | | | |
| --- | --- | --- | --- | --- | --- | --- | --- |
| EARLI | | |  | HOME | | |  |
| Food Item | | Caffeine Content | | Food Item | | Caffeine Content | |
| Coffee | |  | | Coffee | |  | |
|  | Caffeinated coffee | 142 mg/8 fl oz | |  | Instant coffee | 62 mg/8 fl oz | |
|  | Decaffeinated coffee | 2 mg/8 fl oz | |  | Percolated coffee | 95 mg/8 fl oz | |
|  | |  | |  | Drip coffee | 95 mg/8 fl oz | |
|  | |  | |  | Decaffeinated coffee | 2 mg/8 fl oz | |
|  | |  | |  | Espresso | 251 mg/8 fl oz | |
| Tea | |  | | Tea | |  | |
|  | Caffeinated Iced/Hot Tea | 47 mg/8 fl oz | |  | Caffeinated Iced/Hot Tea | 29 mg/8 fl oz | |
|  | Decaffeinated Iced/Hot Tea | 1 mg/8 fl oz | |  | Caffeinated Iced/Hot Tea bags | 29 mg/8 fl oz | |
|  | |  | |  | Herbal Hot/Iced Tea | 0 mg/8 fl oz | |
|  | |  | |  | Decaffeinated Iced/Hot Tea | 2 mg/8 fl oz | |
| Chocolate | |  | | Chocolate | |  | |
|  | Chocolate Candy | 43 mg/ 1.5oz | |  | Hot Chocolate | 5 mg/8 fl oz | |
| Soda | |  | | Soda ^a^ | |  | |
|  | Caffeinated Soda | 33 mg/12 fl oz | |  | Group 1 | 48 mg/12 fl oz | |
|  | Sugar free (Diet) Soda | 33 mg/12 fl oz | |  | Group 2 | 33 mg/12 fl oz | |
|  | Decaffeinated Soda | 0 mg/12 fl oz | |  | Group 3 | 33 mg/12 fl oz | |
|  | |  | |  | Group 4 | 17 mg/12 fl oz | |
|  | |  | |  | Group 5 | 11 mg/12 fl oz | |
| EARLI: Early Autism Risk Longitudinal Investigation Study, HOME: Health Outcomes and Measures of the Environment Study,  ^a^ Groups of soda for HOME:  Group 1:Pepsi One, Mountain Dew, Mellow Yellow, Surge, Kick  Group 2: Pepsi-cola, Coca Cola, Ruby Red, Storm, Big Red, Snapple flavored teas, KMX  Group 3: Diet Soda  Group 4: Canada dry cola, A&W crème soda, Nestea iced tea, Barq’s root beer  Group 5: Mistic tea, Lipton, Brisk tea  ^b^ Study specific caffeine contents were calculated to account for variations in resolution of caffeine intake based on different food frequency questionnaires used between studies. | | | | | | | |

| S2 Table. Unadjusted and Adjusted Differences in Children’s SRS T-Score at Ages 3 to 8 per 1-Unit Increase in mg of Maternal Self-Reported Caffeine Intake During Pregnancy: The EARLI and HOME Studies 2009-2012 and 2003-2006 ^a, b^. | | | |
| --- | --- | --- | --- |
| Cohort/Gestational Period | | Unadjusted | Adjusted |
|  | | β (95% CI) | β (95% CI) |
| EARLI (n=120) | |  |  |
|  | Average | 0.04 (0.01, 0.08) | 0.03 (0.00, 0.07) |
|  | 1^st^ Half (<20 weeks) | 0.03 (0.00, 0.06) | 0.02 (-0.02, 0.05) |
|  | 2^nd^ Half (>20 weeks) | 0.04 (0.01, 0.08) | 0.04 (0.01, 0.08) |
| HOME (n=269) | |  |  |
|  | Average | 0.02 (0.00, 0.05) | 0.01 (-0.01, 0.04) |
|  | 1^st^ Half (<20 weeks) | 0.02 (0.00, 0.04) | 0.01 (-0.01, 0.03) |
|  | 2^nd^ Half (>20 weeks) | 0.01 (-0.01, 0.03) | 0.01 (-0.01, 0.03) |
| EARLI: Early Autism Risk Longitudinal Investigation Study, HOME: Health Outcomes and Measures of the Environment Study, SRS: Social Responsiveness Scale  ^a^ Adjusted for maternal age (continuous), maternal race (white vs non-white), income (<$30,000 vs $30,000-$75,000, ≥ $75,000), parity (continuous), and log_10_ –transformed urine/serum cotinine concentrations (continuous). Note cotinine concentrations were ascertained from maternal urine in EARLI and serum in HOME.  ^b^ Positive coefficients for SRS indicate that maternal caffeine intake is associated with more deficits in social responsiveness traits. | | | |

|  | | | | |  |
| --- | --- | --- | --- | --- | --- |
| S3 Table. Unadjusted and Adjusted Differences in Children’s SRS T-Score at Ages 3 to 8 per IQR Increase in Pooled Maternal Self-Reported Caffeine Intake During Pregnancy: The EARLI and HOME Studies 2009-2012 and 2003-2006 ^a, b^. | | | | |  |
| Cohort/Gestational Period | | IQR ^c^ | Unadjusted | Adjusted |  |
|  | |  | β (95% CI) | β (95% CI) |  |
| Pooled Cohort (n=389) | |  |  |  |  |
|  | Average | 44 | 1.4 (0.5, 2.3) | 1.2 (0.3, 2.0) |  |
|  | 1^st^ Half (<20 weeks) | 50 | 1.1 (0.3, 1.9) | 0.7 (0.1, 1.5) |  |
|  | 2^nd^ Half (>20 weeks) | 41 | 1.0 (0.3, 1.8) | 1.0 (0.2, 1.7) |  |
| EARLI: Early Autism Risk Longitudinal Investigation Study, HOME: Health Outcomes and Measures of the Environment Study, IQR: Interquartile Range, SRS: Social Responsiveness Scale  ^a^ Adjusted for maternal age (continuous), maternal race (white vs non-white), income (<$30,000 vs $30,000-$75,000, ≥ $75,000), parity (continuous), smoking during pregnancy as a binary variable, and cohort. Log_10_ –transformed urine/serum cotinine concentrations (continuous) were used to determine smoking status. Note cotinine concentrations were ascertained from maternal urine in EARLI and serum in HOME.  ^b^ Positive coefficients for SRS indicate that maternal caffeine intake is associated with more deficits in social responsiveness traits.  ^c^ IQR values listed in mg caffeine /day | | | | |  |

| S4 Table. Adjusted Differences in Children’s SRS T-Score at Ages 3 to 8 per IQR Increase Maternal in Self-Reported Caffeine Intake During Pregnancy: The EARLI and HOME Studies, 2009-2012 and 2003-2006 ^a, b, c^. | | | | | | | | | | | | |
| --- | --- | --- | --- | --- | --- | --- | --- | --- | --- | --- | --- | --- |
|  | |  | | | | | |  |  | | | |
| Gestational Period | | EARLI | | |  | | | HOME | | | | |
|  | | IQR ^d^ | β | 95% CI | |  | IQR | | | β | 95% CI |  |
| Primary Analysis | | (n=120) ^e^ |  |  | |  | (n=269) | | |  |  |  |
|  | Average | 57 | 2.0 | (-0.1, 4.0) | |  | 43 | | | 0.6 | (-0.5, 1.6) |  |
|  | 1^st^ Half (<20 weeks) | 66 | 1.0 | (-1.0, 3.0) | |  | 46 | | | 0.4 | (-0.5, 1.3) |  |
|  | 2^nd^ Half (>20 weeks) | 43 | 1.8 | (0.4, 3.2) | |  | 38 | | | 0.3 | (-0.5, 1.1) |  |
| Adjusting for child sex | | (n=120) |  |  | |  | (n=269) | | |  |  |  |
|  | Average | 57 | 2.0 | (-0.1 4.4) | |  | 43 | | | 0.5 | (-0.6, 1.6) |  |
|  | 1^st^ Half (<20 weeks) | 66 | 1.0 | (-1.0, 3.0) | |  | 46 | | | 0.3 | (-0.6, 1.2) |  |
|  | 2^nd^ Half (>20 weeks) | 43 | 1.8 | (0.4, 3.2) | |  | 38 | | | 0.4 | (-0.5, 1.2) |  |
| Adjusting for pre-pregnancy BMI | | (n=119) |  |  | |  | (n=254) | | |  |  |  |
|  | Average | 58 | 2.0 | (-0.0, 4.1) | |  | 43 | | | 0.5 | (-0.6, 1.6) |  |
|  | 1^st^ Half (<20 weeks) | 66 | 1.0 | (-1.0, 3.0) | |  | 46 | | | 0.3 | (-0.6, 1.2) |  |
|  | 2^nd^ Half (>20 weeks) | 66 | 2.9 | (0.7, 5.1) | |  | 38 | | | 0.3 | (-0.6, 1.1) |  |
| Adjusting for total energy intake ^f^ | | (n=120) |  |  | |  |  | | |  |  |  |
|  | Average | 57 | 2.0 | (-0.1, 4.1) | |  | -- | | | -- | -- |  |
|  | 1^st^ Half (<20 weeks) | 66 | 1.3 | (-0.7, 3.3) | |  | -- | | | -- | -- |  |
|  | 2^nd^ Half (>20 weeks) | 43 | 0.7 | (-1.0, 2.3) | |  | -- | | | -- | -- |  |
| Excluding those admitted to NICU ^g^ | |  |  |  | |  | (n=240) | | |  |  |  |
|  | Average | -- | -- | -- | |  | 36 | | | 0.4 | (-0.5, 1.4) |  |
|  | 1^st^ Half (<20 weeks) | -- | -- | -- | |  | 31 | | | 0.3 | (-0.3, 0.9) |  |
|  | 2^nd^ Half (>20 weeks) | -- | -- | -- | |  | 29 | | | 0.2 | (-0.5, 0.8) |  |
| BMI: Body Mass Index, EARLI: Early Autism Risk Longitudinal Investigation Study, HOME: Health Outcomes and Measures of the Environment Study, IQR: Interquartile Range, NICU: Neonatal Intensive Care Unit, SRS: Social Responsiveness Scale  ^a^ Adjusted for maternal age (continuous), maternal race (white vs non-white), income (<$30,000 vs $30,000-$75,000, ≥$75,000), parity (continuous), and log_10_ –transformed urine/serum cotinine concentrations (continuous). Note cotinine concentrations were ascertained from maternal urine in EARLI and serum in HOME. In the pooled cohort model, adjusted for smoking during pregnancy as a binary variable. The pooled results are adjusted for cohort.  ^b^ Positive coefficients for SRS indicate that maternal caffeine intake is associated with more deficits in social responsiveness traits.  ^c^ IQR values listed in mg caffeine /day  ^d^ Note that all beta coefficients and confidence interval values are scaled to the same, full sample IQR.  ^e^ Adjustment for total energy intake was completed using the nutrient residual metho.^57^  ^f^ Note that sample size changes based on data availably by NICU admittance, which was only available within the HOME Study.  ^g^ IQR values of caffeine consumption were updated with respect to changes analytic sample IQRs. | | | | | | | | | | | | |

| S5 Table**.** Adjusted Differences in Children’s SRS T-Score at Ages 3 to 8 per IQR Increase in Maternal Self-Reported Caffeine Intake During Pregnancy from Different Sources of Caffeine: The EARLI and HOME Studies, 2009-2012 and 2003-2006 ^a, b,^ | | | | | | | | | | | | | | | | | |
| --- | --- | --- | --- | --- | --- | --- | --- | --- | --- | --- | --- | --- | --- | --- | --- | --- | --- |
|  | |  |  | | | |  | | |  |  | |  | | |  |  |
| Gestational Period | | IQR ^c,^ | Primary Analysis | |  | Coffee | | |  | Tea | | | |  | Soda | | |
|  | |  | β | 95% CI | | β | | 95% CI | | β | | 95% CI | | | β | | 95% CI |
| EARLI (n=120) | |  |  |  | |  | |  | |  | |  | | |  | |  |
|  | Average | 57 | 2.0 | (-0.1, 4.0) | | 1.5 | | (-0.9, 3.8) | | 4.5 | | (-2.0, 11.0) | | | 0.6 | | (-6.8, 8.1) |
|  | 1^st^ Half (<20 weeks) | 66 | 1.0 | (-1.0, 3.0) | | 0.7 | | (-1.5, 3.0) | | 5.0 | | (-2.1, 12.1) | | | -1.5 | | (-8.0, 5.0) |
|  | 2^nd^ Half (>20 weeks) | 43 | 1.8 | (0.4, 3.2) | | 1.6 | | (-0.2, 3.4) | | 2.5 | | (-1.9, 6.9) | | | 2.1 | | (-2.8, 6.9) |
| HOME (n=269) | |  |  |  | |  | |  | |  | |  | | |  | |  |
|  | Average | 43 | 0.6 | (-0.5, 1.6) | | -0.5 | | (-2.2, 1.2) | | 1.9 | | (-0.5, 4.3) | | | 0.6 | | (-1.0, 2.2) |
|  | 1^st^ Half (<20 weeks) | 46 | 0.4 | (-0.5, 1.3) | | -0.9 | | (-2.3, 0.5) | | 2.2 | | (0.1, 4.2) | | | 0.6 | | (-0.7, 1.8) |
|  | 2^nd^ Half (>20 weeks) | 38 | 0.3 | (-0.5, 1.1) | | 0.2 | | (-1.1, 1.6) | | 0.8 | | (-1.5, 3.2) | | | 0.2 | | (-1.1, 1.4) |
| EARLI: Early Autism Risk Longitudinal Investigation Study, HOME: Health Outcomes and Measures of the Environment Study, IQR: Interquartile Range, SRS: Social Responsiveness Scale  ^a^ Adjusted for maternal age (continuous), maternal race (white vs non-white), income (<$30,000 vs $30,000-$75,000, ≥$75,000), parity (continuous), and log_10_ –transformed urine/serum cotinine concentrations (continuous). Note cotinine concentrations were ascertained from maternal urine in EARLI and serum in HOME. In the pooled cohort model, adjusted for smoking during pregnancy as a binary variable. The pooled results are adjusted for cohort.  ^b^ Positive coefficients for SRS indicate that maternal caffeine intake is associated with more deficits in social responsiveness traits.  ^c^ IQR values listed in mg caffeine /day  ^d^ Note that all beta coefficients and confidence interval values are scaled to the same, full sample IQR. | | | | | | | | | | | | | | | | | |

| S6 Table. Central Tendency and Range of Maternal Self-Reported Estimates of Daily Caffeine Intake Concentrations by Time Period and Caffeine Source. The EARLI and HOME Studies, 2009-2012 and 2003-2006 ^a, b^ | | | | | | | | | | | | | | |
| --- | --- | --- | --- | --- | --- | --- | --- | --- | --- | --- | --- | --- | --- | --- |
| Gestational Period | | All Source Caffeine | | |  | Caffeine from Coffee | |  | Caffeine from Tea | |  | Caffeine from Soda | |  |
|  | | N (%) No Intake ^c^ | Median | 25^th^ & 75^th^ Q | | Median | 25^th^ & 75^th^ Q | | Median | 25^th^ & 75^th^ Q | | Median | 25^th^ & 75^th^ Q | |
| EARLI (n=120) | |  |  |  | |  |  | |  |  | |  |  | |
|  | Average | 2 (2) | 19.5 | (7.7, 65.1) | | 0.1 | (0, 10.2) | | 0.9 | (0, 6.9) | | 0.7 | (0, 6.2) | |
|  | 1^st^ Half (<20 weeks) | 5 (4) | 28.5 | (6.1, 73.3) | | 0 | (0, 15.4) | | 1.0 | (0, 8.1) | | 0.6 | (0, 5.5) | |
|  | 2^nd^ Half (>20 weeks) | 51 (41) | 7.2 | (0, 42.6) | | 0 | (0, 0.9) | | 0 | (0, 3.4) | | 0 | (0, 0.8) | |
| HOME (n=269) | |  |  |  | |  |  | |  |  | |  |  | |
|  | Average | 5 (2) | 18.4 | (5.3, 48.3) | | 0 | (0, 3.8) | | 0 | (0, 3.3) | | 7.2 | (0.3, 20.8) | |
|  | 1^st^ Half (<20 weeks) | 43 (16) | 16.6 | (1.5, 47.0) | | 0 | (0, 1.1) | | 0 | (0, 2.7) | | 4.6 | (0, 18.5) | |
|  | 2^nd^ Half (>20 weeks) | 25 (9) | 15.4 | (3.8, 41.8) | | 0 | (0, 0.8) | | 0 | (0, 0.9) | | 7.6 | (1.0, 18.5) | |
| EARLI: Early Autism Risk Longitudinal Investigation Study, HOME: Health Outcomes and Measures of the Environment Study  ^a^ Daily caffeine intake is estimated as mg caffeine / day  ^b^ Average self-reported caffeine intake is obtained by calculating the mean estimated daily caffeine intake for all participants from the 1^st^ and 2^nd^ halves of pregnancy.  ^c^ Percentage of individuals at each time point who consumed 0 mg of caffeine. | | | | | | | | | | | | | | |

| S7 Table. Adjusted Differences in Children’s SRS T-Score and Raw Scores at Ages 3 to 8 per IQR Increase in Maternal Self-Reported Caffeine Intake During Pregnancy, Stratified by Child Sex: The EARLI and HOME Studies, 2009-2012 and 2003-2006 ^a, b^. | | | | | | | | | | | | |
| --- | --- | --- | --- | --- | --- | --- | --- | --- | --- | --- | --- | --- |
|  | |  |  | | |  | | |  | | |  |
| Gestational Period | | IQR ^c^ | All Children | |  | Females | |  | Males | |  | Sex by Caffeine intake interaction ^e^ |
|  | |  |  |  | |  |  | |  |  | |  |
|  | |  | β | 95% CI | | β | 95% CI | | β | 95% CI | | *P* value |
| EARLI Cohort (n=120) | |  |  |  | |  |  | |  |  | |  |
| SRS T-Scores | |  |  |  | |  |  | |  |  | |  |
|  | Average | 57 | 2.0 | (0.0, 4.0) | | 1.6 | (-1.1, 4.3) | | 2.3 | (-0.8, 5.4) | | 0.54 |
|  | 1^st^ Half (<20 weeks) | 66 | 1.0 | (-1.0, 3.0) | | 1.7 | (-1.1, 4.6) | | 0.7 | (-2.3, 3.6) | | 0.85 |
|  | 2^nd^ Half (>20 weeks) | 43 | 1.9 | (0.5, 3.3) | | 0.9 | (-1.0, 2.87) | | 2.4 | (0.3, 4.4) | | 0.23 |
| SRS Raw Scores | |  |  |  | |  |  | |  |  | |  |
|  | Average | 57 | 4.1 | (-0.4, 8.7) | | 2.9 | (-1.9, 7.8) | | 4.8 | (-2.2, 11.8) | | 0.21 |
|  | 1^st^ Half (<20 weeks) | 66 | 1.9 | (-2.6, 6.4) | | 3.2 | (-1.9, 8.2) | | 1.2 | (-5.5, 7.9) | | 0.13 |
|  | 2^nd^ Half (>20 weeks) | 43 | 4.0 | (0.8, 7.1) | | 1.7 | (-1.7, 5.2) | | 5.1 | (0.4, 9.8) | | 0.24 |
| HOME Cohort (n=269) | |  |  |  | |  |  | |  |  | |  |
| SRS T-Scores | |  |  |  | |  |  | |  |  | |  |
|  | Average | 43 | 0.6 | (-0.5, 1.6) | | 0.7 | (-0.7, 2.1) | | 0.3 | (-1.5, 2.0) | | 0.90 |
|  | 1^st^ Half (<20 weeks) | 46 | 0.4 | (-0.5, 1.3) | | 0.6 | (-0.4, 1.7) | | -1.2 | (-3.2, 0.8) | | 0.46 |
|  | 2^nd^ Half (>20 weeks) | 38 | 0.3 | (-0.5, 1.1) | | 0.1 | (-1.1, 1.4) | | 0.6 | (-0.5, 1.6) | | 0.40 |
| SRS Raw Scores | |  |  |  | |  |  | |  |  | |  |
|  | Average | 57 | 1.0 | (-1.2, 3.1) | | 1.3 | (-1.4, 3.9) | | 0.6 | (-3.2, 4.4) | | 0.11 |
|  | 1^st^ Half (<20 weeks) | 66 | 0.4 | (-1.3, 2.1) | | 1.2 | (-0.8, 3.1) | | -2.5 | (-6.8, 1.8) | | 0.06 |
|  | 2^nd^ Half (>20 weeks) | 43 | 0.8 | (-0.8, 2.4) | | 0.2 | (-2.1, 2.6) | | 1.3 | (-0.9, 3.5) | | 0.12 |
| EARLI: Early Autism Risk Longitudinal Investigation Study, HOME: Health Outcomes and Measures of the Environment Study, IQR: Interquartile Range, SRS: Social Responsiveness Scale  ^a^ Adjusted for maternal age (continuous), maternal race (white vs non-white), income (<$30,000 vs $30,000-$75,000, ≥$75,000), parity (continuous), and log_10_ –transformed urine/serum cotinine concentrations (continuous). Note cotinine concentrations were ascertained from maternal urine in EARLI and serum in HOME. In the pooled cohort model, adjusted for smoking during pregnancy as a binary variable. The pooled results are adjusted for cohort.  ^b^ Positive coefficients for SRS indicate that maternal caffeine intake is associated with more deficits in social responsiveness traits.  ^c^ IQR values listed in mg caffeine /day  ^d^ Note that all beta coefficients and confidence interval values are scaled to the same, full sample IQR.  ^e^ Child sex by maternal caffeine interaction terms used to assess significance of effect measure modification between female and male children for the association between maternal caffeine intake and SRS T-scores. Note that *P* value is 2 sided. | | | | | | | | | | | | |

| S8 Table. Adjusted Differences in Children’s SRS Subscale T-Score at Ages 3 to 8 per IQR Increase in Maternal Self-Reported Caffeine Intake During Pregnancy: The EARLI and HOME Studies, 2009-2012 and 2003-2006 ^a, b^. | | | | | | | | | | | | | | | | | | | | | | | | | |
| --- | --- | --- | --- | --- | --- | --- | --- | --- | --- | --- | --- | --- | --- | --- | --- | --- | --- | --- | --- | --- | --- | --- | --- | --- | --- |
|  | |  |  | | | |  | | | |  | |  |  |  | | | | | |  | |  | | |
|  | |  | Primary Analysis | |  | Awareness | |  | Cognition | | |  | Communication | | | |  | Mannerism | |  | | Motivation | | |  |
| Gestational Period | | IQR ^c^ | β | 95% CI | | β | 95% CI | | β | 95% CI | | | β | | | 95% CI | | β | 95% CI | | | β | | 95% CI | |
| EARLI (n=120) | |  |  |  | |  |  | |  |  | | |  | | |  | |  |  | | |  | |  | |
|  | Average | 57 | 2.0 | (-0.1, 4.0) | | 2.0 | (0.0, 4.0) | | 0.9 | (-0.8, 2.7) | | | 2.0 | | | (-0.2, 4.1) | | 1.3 | (-0.9, 3.4) | | | 1.3 | | (-0.7, 3.3) | |
|  | 1^st^ Half (<20 weeks) | 66 | 1.0 | (-1.0, 3.0) | | 1.0 | (-1.0, 2.9) | | 1.1 | (-0.9, 3.1) | | | 0.9 | | | (-1.3, 3.1) | | 0.3 | (-1.8, 2.3) | | | 0.6 | | (-1.4, 2.6) | |
|  | 2^nd^ Half (>20 weeks) | 43 | 1.8 | (0.4, 3.2) | | 1.9 | (0.5, 3.2) | | 1.6 | (0.2, 3.0) | | | 1.9 | | | (0.4, 3.4) | | 1.5 | (0.0, 2.9) | | | 1.3 | | (-0.1, 2.6) | |
| HOME (n=269) | |  |  |  | |  |  | |  |  | | |  | | |  | |  |  | | |  | |  | |
|  | Average | 43 | 0.6 | (-0.5, 1.6) | | 0.3 | (-0.8, 1.4) | | 0.2 | (-1.0, 1.4) | | | 0.6 | | | (-0.5, 1.7) | | 1.0 | (-0.1, 2.2) | | | 0.2 | | (-1.0, 1.2) | |
|  | 1^st^ Half (<20 weeks) | 46 | 0.4 | (-0.5, 1.3) | | -0.3 | (-1.7, 1.2) | | -1.1 | (-2.6, 0.4) | | | -0.9 | | | (-2.3, 0.5) | | -0.4 | (-1.9, 1.4) | | | -0.7 | | (-2.1, 0.7) | |
|  | 2^nd^ Half (>20 weeks) | 38 | 0.3 | (-0.5, 1.1) | | 0.6 | (-0.8, 1.9) | | -0.3 | (-1.7, 1.1)` | | | 0.4 | | | (-0.9, 1.7) | | 0.0` | (-1.4, 1.4) | | | 0.3 | | (-1.1, 1.6) | |
| EARLI: Early Autism Risk Longitudinal Investigation Study, HOME: Health Outcomes and Measures of the Environment Study, IQR: Interquartile Range, SRS: Social Responsiveness Scale  ^a^ Adjusted for maternal age (continuous), maternal race (white vs non-white), income (<$30,000 vs $30,000-$75,000, ≥$75,000), parity (continuous), and log_10_ –transformed urine/serum cotinine concentrations (continuous). Note cotinine concentrations were ascertained from maternal urine in EARLI and serum in HOME. In the pooled cohort model, adjusted for smoking during pregnancy as a binary variable. The pooled results are adjusted for cohort.  ^b^ Positive coefficients for SRS indicate that maternal caffeine intake is associated with more deficits in social responsiveness traits.  ^c^ IQR values listed in mg caffeine /day  ^d^ Note that all beta coefficients and confidence interval values are scaled to the same, full sample IQR. | | | | | | | | | | | | | | | | | | | | | | | | | |

|  | | | | | |
| --- | --- | --- | --- | --- | --- |
| S9 Table. Unadjusted and Adjusted Modified Poisson Model, Estimating the RR of Children’s SRS T-Score ≥ 60 and ≥75 at Ages 3 to 8 per IQR Increase in Maternal Self-Reported Caffeine Intake During Pregnancy: The EARLI and HOME Studies, 2009-2012 and 2003-2006 ^a, b^. | | | | | |
| Gestational Period | |  | Unadjusted | Adjusted: SRS T-score ≥ 60 ^d^ | Adjusted: SRS T-score ≥75 ^e^ |
|  | | IQR ^c^ | RR (95% CI) | RR (95% CI) | RR (95% CI) |
| EARLI (n=120) | |  |  |  |  |
|  | Average | 57 | 1.2 (0.9, 1.8) | 1.1 (0.7, 1.8) | 1.3 (0.8, 2.3) |
|  | 1^st^ Half (<20 weeks) | 66 | 1.1 (0.8, 1.7) | 0.9 (0.5, 1.4) | 0.9 (0.5, 1.8) |
|  | 2^nd^ Half (>20 weeks) | 43 | 1.2 (0.9, 1.6) | 1.3 (0.9, 1.8) | 1.5 (1.0, 2.2) |
| HOME (n=269) | |  |  |  |  |
|  | Average | 43 | 1.2 (0.9, 1.5) | 1.1 (0.8, 1.5) | 1.4 (0.9, 2.4) |
|  | 1^st^ Half (<20 weeks) | 46 | 1.1 (0.9, 1.4) | 1.1 (0.8, 1.3) | 1.3 (0.9, 1.8) |
|  | 2^nd^ Half (>20 weeks) | 38 | 1.1 (0.9, 1.3) | 1.1 (0.9, 1.4) | 1.3 (0.9, 1.8) |
| EARLI: Early Autism Risk Longitudinal Investigation Study, HOME: Health Outcomes and Measures of the Environment Study, IQR: Interquartile Range, RR: Relative Risk, SRS: Social Responsiveness Scale  ^a^ Adjusted for maternal age (continuous), maternal race (white vs non-white), income (<$30,000 vs $30,000-$75,000, ≥$75,000), parity (continuous), and log_10_ –transformed urine/serum cotinine concentrations (continuous). Note cotinine concentrations were ascertained from maternal urine in EARLI and serum in HOME.  ^b^ Positive coefficients for SRS indicate that maternal caffeine intake is associated with more deficits in social responsiveness traits.  ^c^ IQR values listed in mg caffeine /day.  ^d^ EARLI: SRS t-scores ≥60 (n=20); HOME: SRS t-scores ≥60 (n=43).  ^e^ EARLI: SRS t-scores ≥75 (n=10); HOME: SRS t-scores ≥75 (n=11). | | | | | |

| S10 Table. Adjusted Differences in Children’s SRS T-Score at Ages 3 to 8 per IQR Increase in Maternal Self-Reported Caffeine Intake During Pregnancy Excluding Cotinine in Adjusted Analyses: The EARLI and HOME Studies, 2009-2012 and 2003-2006 ^a^. | | | | | | | |
| --- | --- | --- | --- | --- | --- | --- | --- |
| Gestational Period | | IQR ^b^ | Primary Analysis | |  | Adjusted Analysis, excluding cotinine ^c^ | |
|  | |  | β | 95% CI | | β | 95% CI |
| EARLI (n=120) | |  |  |  | |  |  |
|  | Average | 57 | 2.0 | (-0.1, 4.0) | | 2.6 | (0.5, 4.6) |
|  | 1^st^ Half (<20 weeks) | 66 | 1.0 | (-1.0, 3.0) | | 1.7 | (-0.4, 3.7) |
|  | 2^nd^ Half (>20 weeks) | 43 | 1.8 | (0.4, 3.2) | | 2.2 | (0.7, 3.6) |
| HOME (n=269) | |  |  |  | |  |  |
|  | Average | 43 | 0.6 | (-0.5, 1.6) | | 0.5 | (-0.5, 1.6) |
|  | 1^st^ Half (<20 weeks) | 46 | 0.4 | (-0.5, 1.3) | | 0.4 | (-0.4, 1.2) |
|  | 2^nd^ Half (>20 weeks) | 38 | 0.3 | (-0.5, 1.1) | | 0.3 | (-0.4, 1.0) |
| EARLI: Early Autism Risk Longitudinal Investigation Study, HOME: Health Outcomes and Measures of the Environment Study, IQR: Interquartile Range, RR: Relative Risk, SRS: Social Responsiveness Scale  ^a^ Adjusted for maternal age (continuous), maternal race (white vs non-white), income (<$30,000 vs $30,000-$75,000, ≥$75,000), parity (continuous), and log_10_ –transformed urine/serum cotinine concentrations (continuous). Note cotinine concentrations were ascertained from maternal urine in EARLI and serum in HOME.  ^b^ IQR values listed in mg caffeine /day  ^c^ Adjusted for maternal age (continuous), maternal race (white vs non-white), income (<$30,000 vs $30,000-$75,000, ≥$75,000), and parity (continuous) | | | | | | | |

| S11 Table. Adjusted Differences in Children’s SRS T-Score at Ages 3 to 8 per IQR Increase in Maternal Self-Reported Caffeine Intake During Pregnancy Stratified by Maternal Pre-Pregnancy BMI category: The EARLI and HOME Studies 2009-2012 and 2003-2006^a, b^. | | | | | | | | | | | | | | | |
| --- | --- | --- | --- | --- | --- | --- | --- | --- | --- | --- | --- | --- | --- | --- | --- |
|  | |  |  |  | | |  |  | |  |  | |  | |  |
|  | |  |  | Normal BMI ^e^ | |  | Overweight BMI | | |  | Obese BMI | | |  | BMI x Caffeine interaction |
|  | |  |  |  |  | |  | |  | |  |  | | |  |
| Gestational Period | |  | IQR ^c^ | β | 95% CI | | β | | 95% CI | | β | 95% CI | | | P-value |
| EARLI (n=119) ^d^ | |  |  |  |  | |  | |  | |  |  | | |  |
|  | Average |  | 58 | 1.8 | (-2.6, 6.2) | | 1.0 | | (-2.7, 4.7) | | 1.6 | (-2.3, 5.5) | | | 0.915 |
|  | 1^st^ Half (<20 weeks) |  | 66 | 0.9 | (-3.3, 5.1) | | 0.8 | | (-3.0, 4.7) | | 0.2 | (-3.2, 3.6) | | | 0.944 |
|  | 2^nd^ Half (>20 weeks) |  | 66 | 2.1 | (-2.3, 6.6) | | 1.4 | | (-3.0, 5.7) | | 3.0 | (-1.2, 7.3) | | | 0.864 |
| HOME (n=254) | |  |  |  |  | |  | |  | |  |  | | |  |
|  | Average |  | 43.2 | -0.5 | (-2.1, 1.1) | | 0.8 | | (-1.3, 2.9) | | 2.9 | (-0.0, 5.8) | | | 0.039 |
|  | 1^st^ Half (<20 weeks) |  | 45.8 | -0.4 | (-1.6, 0.9) | | 0.5 | | (-1.1, 2.1) | | 2.1 | (-0.1, 4.2) | | | 0.023 |
|  | 2^nd^ Half (>20 weeks) |  | 41.2 | -0.3 | (-1.4, 0.9) | | 0.5 | | (-1.1, 2.2) | | 1.9 | (-1.2, 4.9) | | | 0.142 |
| BMI: Body Mass Index, EARLI: Early Autism Risk Longitudinal Investigation Study, HOME: Health Outcomes and Measures of the Environment Study, IQR: Interquartile Range, SRS: Social Responsiveness Scale  ^a^ Adjusted for maternal age (continuous), maternal race (white vs non-white), income (<$30,000 vs $30,000-$75,000, ≥$75,000), parity (continuous), and log_10_ –transformed urine/serum cotinine concentrations (continuous). Note cotinine concentrations were ascertained from maternal urine in EARLI and serum in HOME. In the pooled cohort model, adjusted for smoking during pregnancy as a binary variable. The pooled results are adjusted for cohort.  ^b^ Positive coefficients for SRS indicate that maternal caffeine intake is associated with more deficits in social responsiveness traits.  ^c^ IQR values listed in mg caffeine /day  ^d^ Note that all beta coefficients and confidence interval values are scaled to the same, full sample IQR.  ^e^ Note that pre-pregnancy BMI information was only available for a subset of each sample (EARLI n=119; HOME n=259)  ^f^ Normal/Underweight: BMI <25, Overweight: BMI ≥25<30, and Obese BMI≥30 | | | | | | | | | | | | | | | |

**References**

43. USDA Food Composition Database | National Agricultural Library | USDA. Agricultural Research Service Food Composition Database. Accessed June 12, 2020. https://www.nal.usda.gov/usda-food-composition-database

57. Willett WC, Howe GR, Kushi LH. Adjustment for total energy intake in epidemiologic studies. *Am J Clin Nutr*. 1997;65(4 Suppl):1220S-1228S; discussion 1229S-1231S. doi:10.1093/ajcn/65.4.1220S

81. The Tea Association of The USA. Tea Council of the USA. INC. Published 2011. Accessed June 12, 2020. <http://www.teausa.com/>

82. U.S. coffee consumption share by type of coffee, 2019. Statista. Accessed June 12, 2020. https://www.statista.com/statistics/250064/us-roasted-coffee-consumption-by-type-of-coffee/
