# Supplementary figures and images for: Association between self-reported caffeine intake during pregnancy and social responsiveness scores in childhood: The EARLI and HOME studies
Source: PLoS One. 2021 Jan 15;16(1):e0245079. doi: 10.1371/journal.pone.0245079 (PMC7810310; doi:10.1371/journal.pone.0245079)

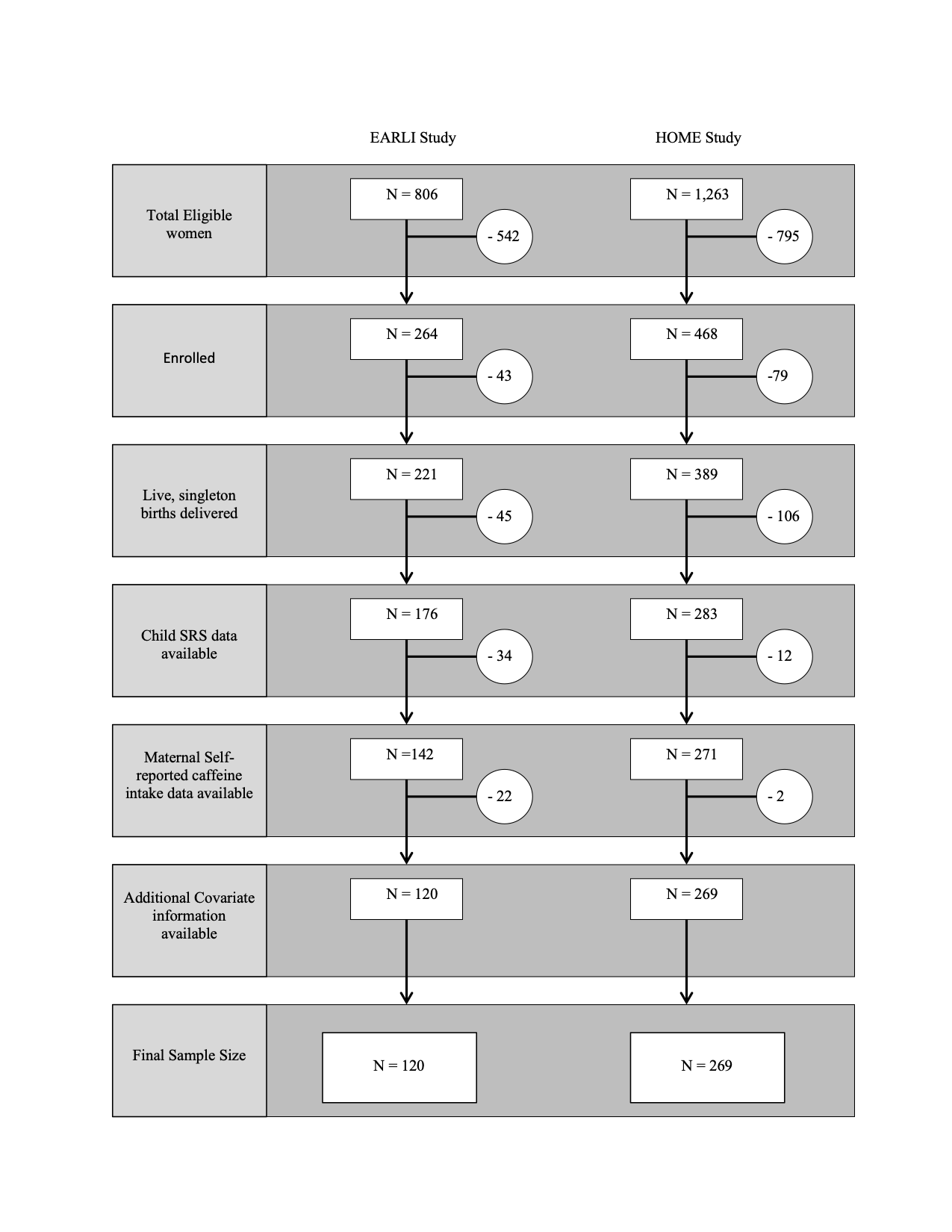

Supplement: S1 Fig — (TIF) [file pone.0245079.s002.tif]

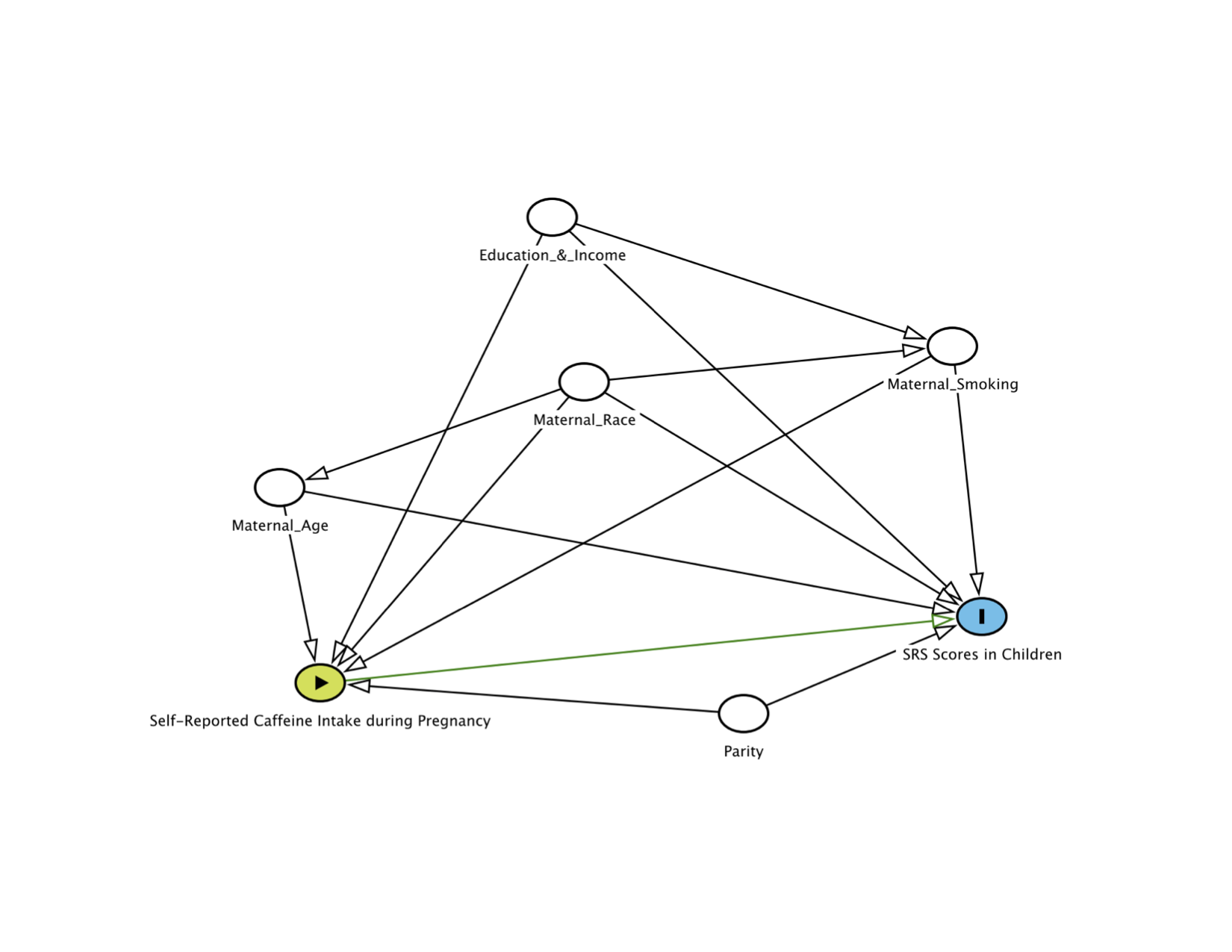

Supplement: S2 Fig — (TIF) [file pone.0245079.s003.tif]

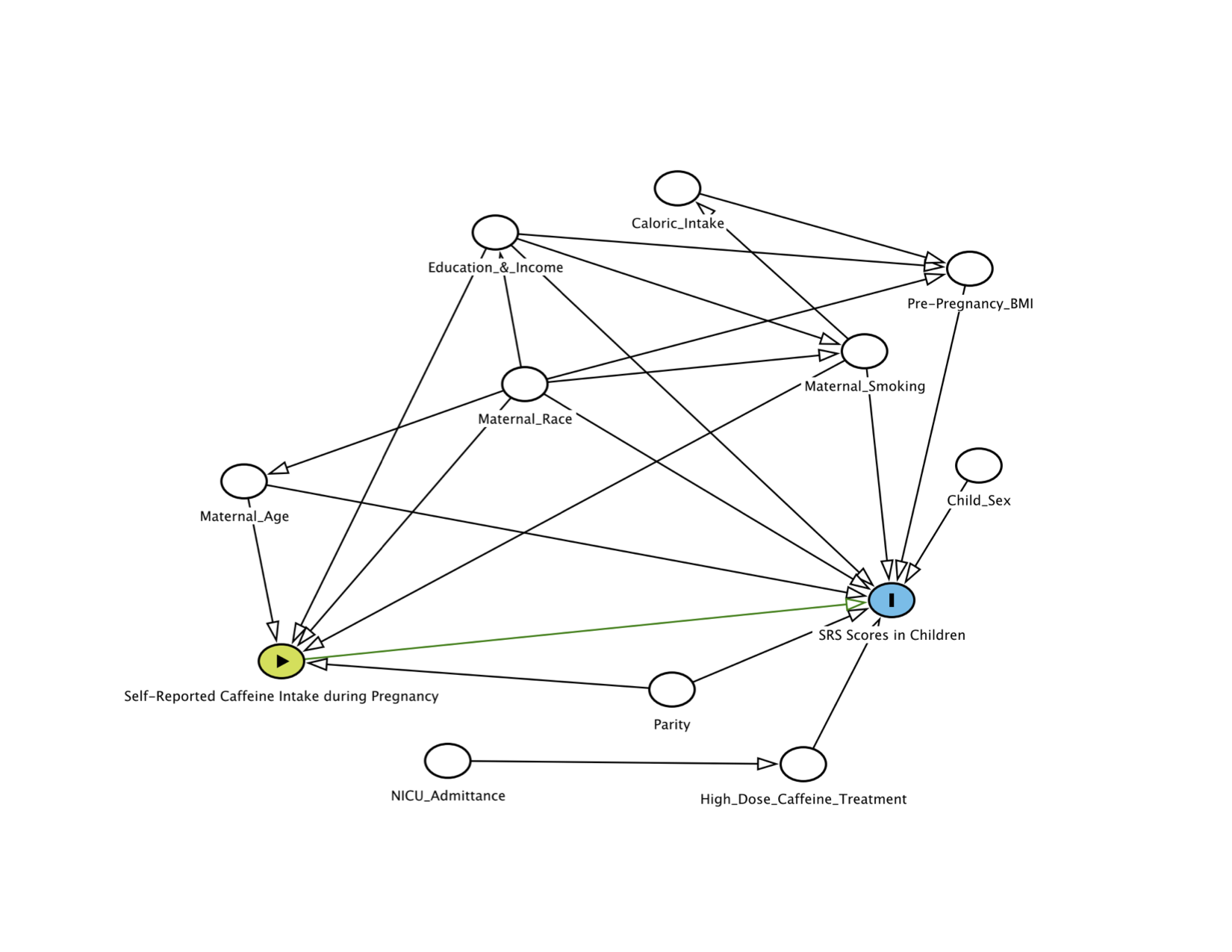

Supplement: S3 Fig — (TIF) [file pone.0245079.s004.tif]

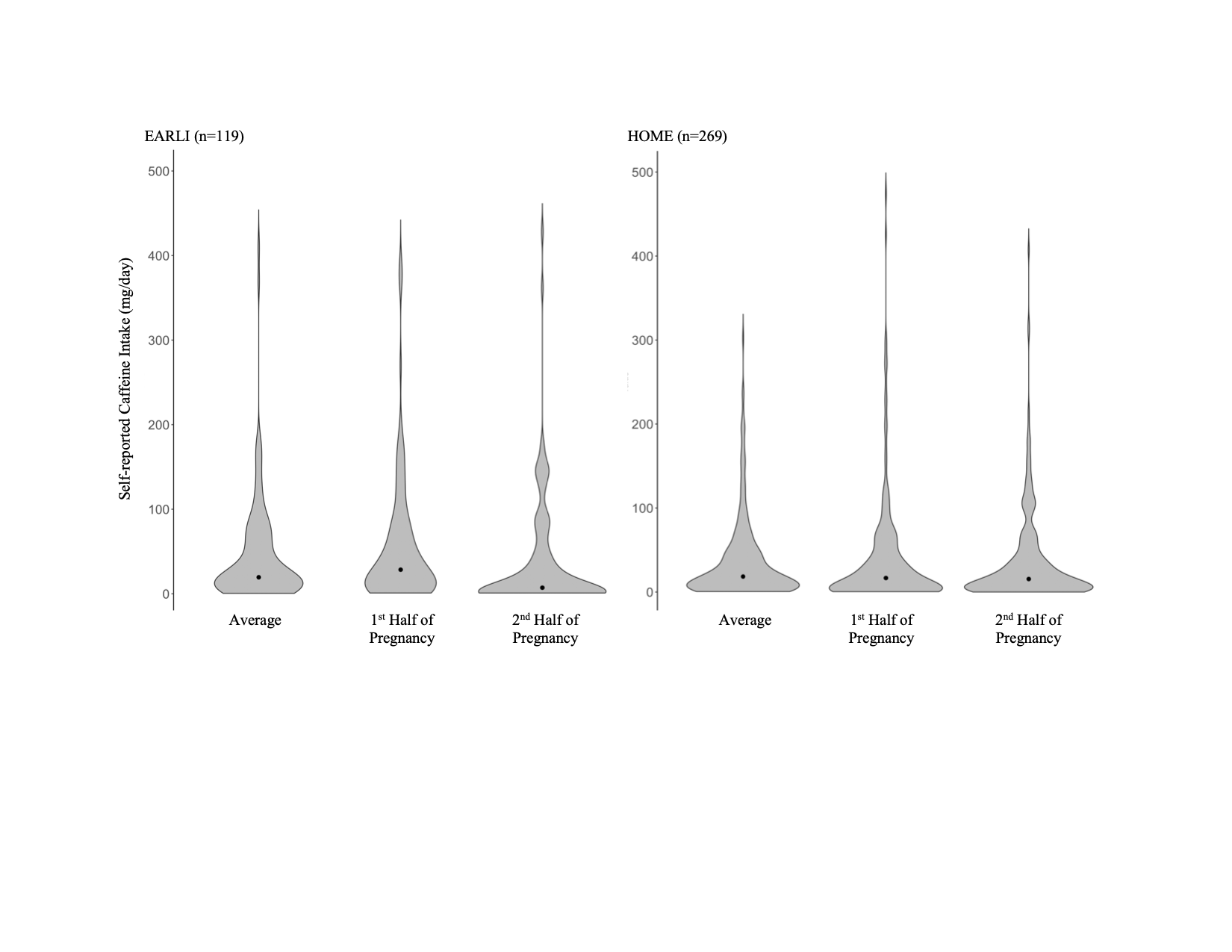

Supplement: S4 Fig — a Median values of self-reported caffeine intake for each time period have been marked with a dark circle. Median values for maternal self-reported caffeine intake for EARLI: Average: 20mg/day, 1st half of pregnancy: 28mg/day, 2nd half of pregnancy: 7 mg/day; HOME: Average: 18 mg/day, 1st half of pregnancy:17mg/day, 2nd half of pregnancy: 15mg/day b Average caffeine intake values were estimated from the 1st and 2nd halves of pregnancy exposure measures. c Each graph shown is a density function, and represents the distribution of maternal self-reported caffeine intake at each time point. (TIF) [file pone.0245079.s005.tif]

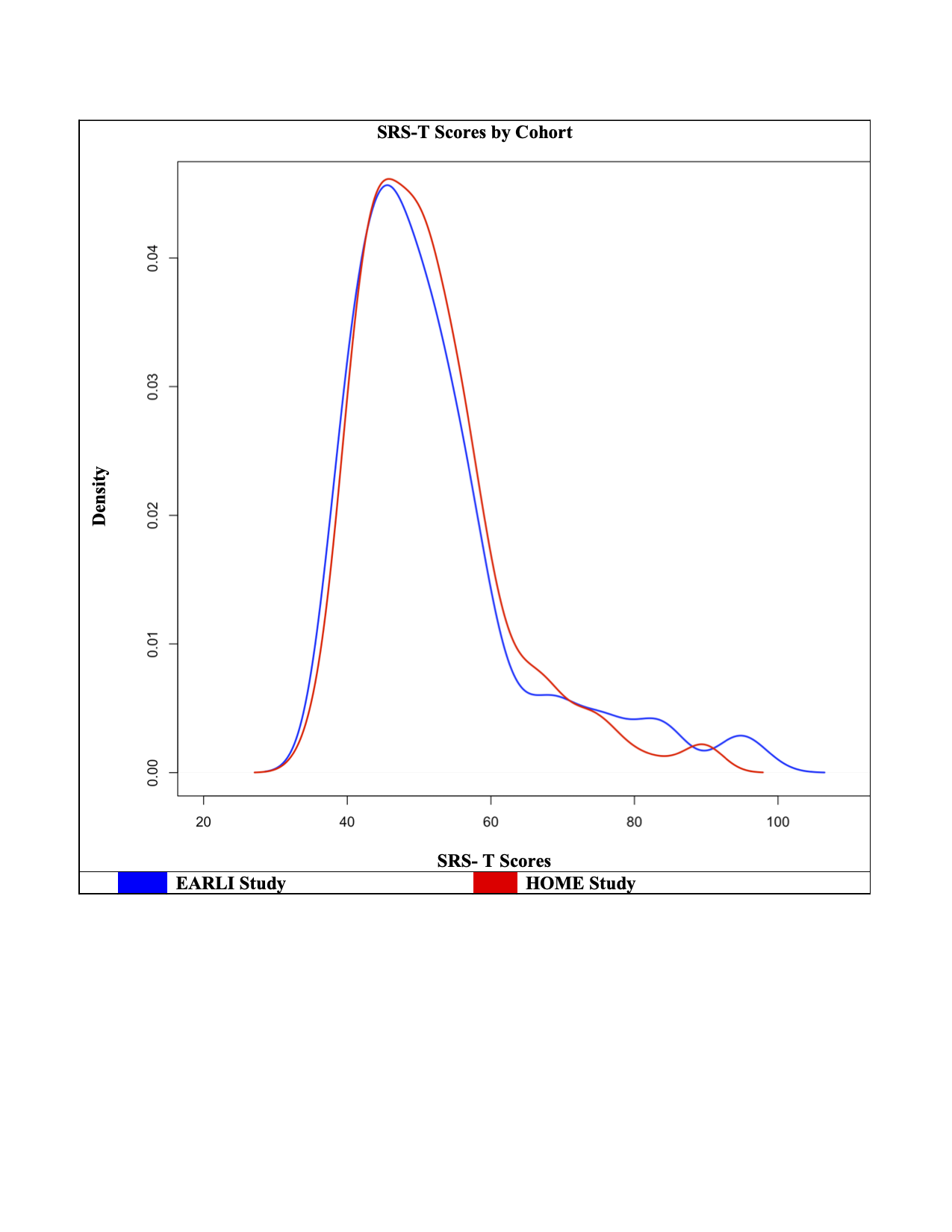

Supplement: S5 Fig — a The central tendencies of child SRS T-scores were similar in EARLI (mean: 52, SD: 13) and HOME (mean: 51, SD: 10). b SRS T-scores ranging from 60–75 are indicative of clinically significant deficiencies in reciprocal social behavior that may interfere with daily social interactions, while scores greater than 75 are strongly associated with clinical diagnosis of ASD. (TIF) [file pone.0245079.s006.tif]

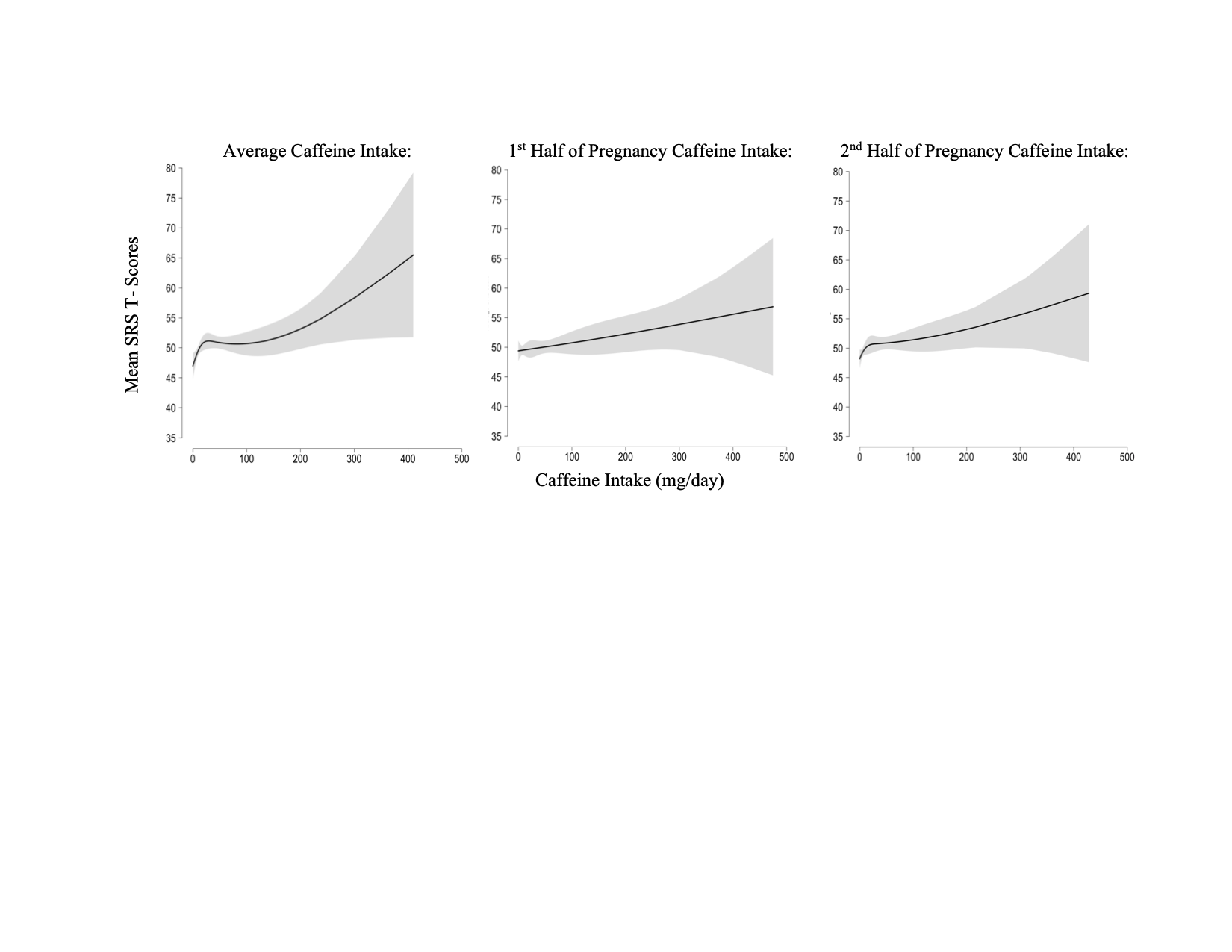

Supplement: S6 Fig — a Adjusted for maternal age (continuous), maternal race (white vs non-white), income (<$30,000 vs $30,000-$75,000, ≥$75,000), parity (continuous), smoking during pregnancy as a binary variable, and cohort. Log10 –transformed urine/serum cotinine concentrations (continuous) were used to determine smoking status. Note cotinine concentrations were ascertained from maternal urine in EARLI and serum in HOME. (TIF) [file pone.0245079.s007.tif]
